# Thermal reactionomes reveal divergent responses to thermal extremes in warm and cool-climate ant species

**DOI:** 10.1186/s12864-016-2466-z

**Published:** 2016-03-02

**Authors:** John Stanton-Geddes, Andrew Nguyen, Lacy Chick, James Vincent, Mahesh Vangala, Robert R. Dunn, Aaron M. Ellison, Nathan J. Sanders, Nicholas J. Gotelli, Sara Helms Cahan

**Affiliations:** Department of Biology, University of Vermont, Burlington, VT 05405 USA; Department of Ecology and Evolutionary Biology, University of Tennessee, Knoxville, TN 37996 USA; Vermont Genetics Network, University of Vermont, Burlington, VT 05405 USA; Department of Biological Sciences, North Carolina State University, Raleigh, NC 27695 USA; Harvard Forest, Harvard University, Petersham, MA 01336 USA; Center for Macroecology, Evolution and Climate, University of Copenhagen, Universitetsparken 15, DK-2100 Copenhagen, Denmark; Data Scientist, Dealer.com, 1 Howard St, Burlington, VT 05401 USA

**Keywords:** *Aphaenogaster*, Gene expression, Plasticity, Reactionome, Transcriptome

## Abstract

**Background:**

The distributions of species and their responses to climate change are in part determined by their thermal tolerances. However, little is known about how thermal tolerance evolves. To test whether evolutionary extension of thermal limits is accomplished through enhanced cellular stress response (*enhanced response*), constitutively elevated expression of protective genes (*genetic assimilation*) or a shift from damage resistance to passive mechanisms of thermal stability (*tolerance*), we conducted an analysis of the *reactionome*: the reaction norm for all genes in an organism’s transcriptome measured across an experimental gradient. We characterized thermal reactionomes of two common ant species in the eastern U.S, the northern cool-climate *Aphaenogaster picea* and the southern warm-climate *Aphaenogaster carolinensis*, across 12 temperatures that spanned their entire thermal breadth.

**Results:**

We found that at least 2 % of all genes changed expression with temperature. The majority of upregulation was specific to exposure to low temperatures. The cool-adapted *A. picea* induced expression of more genes in response to extreme temperatures than did *A. carolinensis*, consistent with the *enhanced response* hypothesis. In contrast, under high temperatures the warm-adapted *A. carolinensis* downregulated many of the genes upregulated in *A. picea*, and required more extreme temperatures to induce down-regulation in gene expression, consistent with the *tolerance* hypothesis. We found no evidence for a trade-off between constitutive and inducible gene expression as predicted by the *genetic assimilation hypothesis*.

**Conclusions:**

These results suggest that increases in upper thermal limits may require an evolutionary shift in response mechanism away from damage repair toward tolerance and prevention.

**Electronic supplementary material:**

The online version of this article (doi:10.1186/s12864-016-2466-z) contains supplementary material, which is available to authorized users.

## Background

Temperature regulates biological activity and shapes diversity from molecular to macroecological scales [1, 2]. Many species, especially small-bodied arthropods, live at temperatures close to their thermal limits and are at risk from current increases in temperature [3–5]. Thermal tolerance, the ability of individuals to maintain function and survive thermal extremes, depends on a complex interplay between the structural integrity of cellular components and activation of physiological response mechanisms to prevent and/or repair damage [6, 7]. Thermal defense strategies are shaped by the environmental regime organisms experience [8] and thermal limits vary considerably among species and populations [3, 4, 9, 10]. These differences in thermal tolerance are largely genetic [11, 12] with a highly polygenic basis [13–16]. Outside of candidate genes [13], little is known about the evolution of thermal tolerance or the link between short-term physiological acclimation and longer-term adaptation to novel temperature regimes. This information is critical for understanding the adaptive potential of species to future climates [17].

To address this gap of knowledge, we need information on the extent to which selection has acted upon the diversity and plasticity of genes involved in thermal tolerance [17, 18]. In recent years, whole-organism gene expression approaches (e.g. transcriptomics) using high-throughput RNA sequencing (RNAseq) technology have been widely applied to identify genes involved in thermal tolerance [19–22] and other traits. Such studies typically use an ANOVA-type experimental or sampling design, with only a few environmental levels, and often find only a few dozen to hundred genes with differential expression in different thermal regimes. However, temperature and other environmental factors vary continuously in nature. As a result, such categorical comparisons (e.g. high vs. low temperatures) are likely to miss key differences that are due not just to whether it is hot, but rather how hot it is. Continuous variation is better characterized with a reaction norm approach, which describes variation in the phenotype of a single genotype across an environmental gradient [23]. Reaction norms differ not only in mean values, but also in their shapes [10, 24], and differences in the shape of reaction norms are often larger than differences in mean values at both the species and the population level [24].

In this study, we extend the reaction norm approach to RNAseq analysis and introduce the *reactionome*, which we define as a characterization of the reaction norm for all genes in an organism’s transcriptome across an environmental gradient. Although temporal patterns of transcriptional activity (e.g. fast- vs. slow- responding genes) are also important components of an organism’s transcriptional response to environmental conditions [25], we focus here on the response of transcripts across conditions at the same time point.

We use the reactionome method to identify genes that are thermally responsive in two closely-related eastern North American ant species, *Aphaenogaster carolinensis* and *A. picea* [26, 27]. *Aphaenogaster* are some of the most common ants in eastern North America [28] where they are keystone seed dispersers [29–31]. Ants, and ecotherms in general, have little or no thermal safety margin [5] and thus are highly susceptible to climate change [4, 32], putting at risk important ecosystem services [33]. Growth chamber studies have demonstrated that reproduction of *Aphaenogaster* will be compromised by increased temperatures [34], while field studies [32] and simulations [35] indicate that ant species persistence will depend on combinations of physiology and species interactions. *Aphaenogaster carolinensis* experiences a higher mean annual temperature (MAT) (14.6 °C) and less seasonal temperature variation (temperature seasonality = 7678°) than does *A. picea* (MAT = 4.6 °C, seasonality = 10,008°; [36]) at their respective collection sites. In controlled laboratory experiments, these warm- and cold-climate species exhibit corresponding differences in their critical maximum (44.7 °C for *A. carolinensis* versus 41.3 °C for *A. picea*; see Methods) and minimum temperatures (6.1 °C for *A. carolinensis* versus −0.1 °C for *A. picea*). These differences between species in their thermal environments and physiological tolerances allowed us to investigate adaptation to both lower and upper thermal extremes in this system.

To characterize the thermal reactionome, we measured the reaction norm for each gene using a regression-based statistical approach to identify temperature-dependent patterns of change in gene expression. We used these response patterns to quantitatively test three mechanistic hypotheses of thermal adaptation. First, the *enhanced response hypothesis* [37–39] proposes that species extend their thermal limits through a stronger induced response to provide greater protection from more frequently encountered stressors. This hypothesis would predict that the cool-adapted *A. picea* would activate more genes, and induce them more strongly, in response to low temperatures than would the warm-adapted *A. carolinensis*, which would show greater induction in response to high temperatures.

Second, the *tolerance hypothesis* [9, 40] proposes that existing inducible stress responses become insufficient or prohibitively costly as environmental stressors increase in frequency, resulting in a shift away from induced response in favor of structural changes [41] or behavioral adaptations [5, 42]. This hypothesis predicts adaptation to stress should be associated with lower transcriptional responsiveness and less sensitivity to temperature perturbation, as well as a shift to an alternate suite of tolerance genes and pathways [43, 44].

Finally, the *genetic assimilation hypothesis* [45, 46] proposes that exposure to more extreme stressors selects for a shift from inducible to constitutive expression of stress-response genes. This hypothesis predicts that transcripts responsive to high temperatures in *A. picea* will have higher constitutive expression in *A. carolinensis*, whereas transcripts responsive to low temperatures in *A. carolinensis* will have higher constitutive expression in *A. picea*. Of course, these hypotheses are not mutually exclusive. By taking a reactionome approach, we can quantify if, and under what conditions, these mechanisms contribute to thermal adaptation.

To summarise, in this project we generated the transcriptomes of two closely-related temperate ant species, and quantified their gene expression across a wide range of thermal conditions. We then evaluated three non-mutually exclusive hypotheses (enhanced response, tolerance and genetic assimilation) of the evolution of thermal adaptation by comparing the number and expression patterns of transcripts between species in response to extreme low and extreme high temperatures. Finally, we used gene ontology information to determine which gene products and pathways are involved in thermal adaptation in the two species.

## Results

### Reaction norms of thermally-responsive transcripts

The combined *Aphaenogaster* transcriptome assembly contained 99,861 transcripts. About half of these (51,246) transcripts had a signficant BLAST hit, of which 50 % (25,797) had a top hit to Insecta and 37 % (18,854) had a top hit to Formicidae. We performed a BUSCO analysis [47] to assess the quality of the transcriptome assembly against the arthropod Benchmarking Universal Single-Copy Orthologs (BUSCOs). This analysis revealed that the transcriptome is largely complete, as we recovered 1,426 complete single-copy BUSCOs (62%) and an additional 435 fragmented BUSCOs (16%), which is in line with the results of Simao et al [47] for transcriptomes of other non-model species. Moreover, only 8% of the BUSCOs were found to be duplicated in the combined transcriptome, which indicates that the steps (see Methods) we took to collapse homologs between the two species in the transcriptome assembly were successful.

We quantified gene expression using the program Sailfish [48], and fitted polynomial regression models to the expression values of each transcript to identify those that had a linear or quadratic relationship. To account for multiple tests, we both applied a False Discovery Rate (FDR) correction, and performed a resampling analysis to determine the number of transcripts that would be expected to have a significant relationshp by chance alone. We retained the 2509 (2.5 % of total) true positive transcripts that exceeded null expectation from the resampling analysis for further analysis (Additional file 1: Table S1). Of these transcripts, 75 % (1553) had a non-linear relationship with temperature that would likely have been missed with a standard differential expression experiment (e.g. high vs. low temperature). The proportion of responsive transcripts is similar if we focus only on those transcript with a BLAST hit (725 significant transcripts out of 51,246). However, as with all *de novo* transcriptome assemblies, this assembly is fragmented due to partial contigs and alternative transcripts [49] so this estimate is likely a lower bound for the true proportion of transcripts that are thermally responsive.

**Fig. 1:**
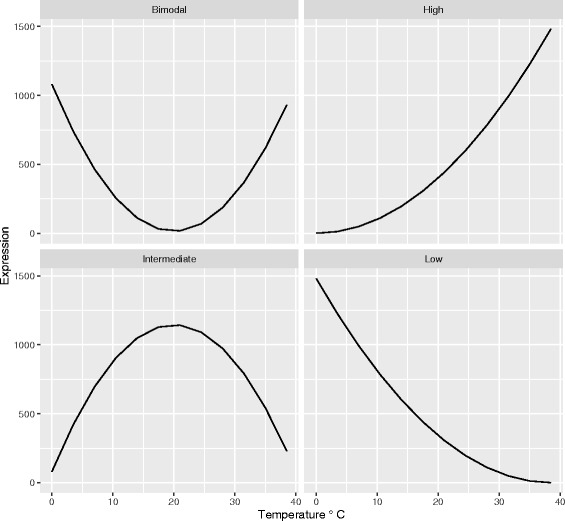
Illustrations of the pattern in relation to temperature for each of the four expression categories; Bimodal, High, Intermediate and Low. The fifth category of Not Responsive (a flat line) is not shown

We used the predicted transcript expression levels to partition transcripts for each species into five expression categories (Fig. 1) which were defined *a priori* to allow us to test predictions derived from three thermal adaptation hypotheses of relative response severity in the two species: **High** transcripts had greatest expression at temperatures > 31 °C, **Low **transcripts had greatest expression at temperatures < 10 °C, **Intermediate** transcripts had greatest expression between 10 to 30 °C, **Bimodal** transcripts had increased expression at both high and low temperatures, while **NotResp** transcripts were those that were not thermally responsive in the focal species but did respond in the other.

### Expression response to thermal extremes differs between species

Although the total number of thermally-responsive transcripts did not differ between species (χ^2^_1_ = 0.08, *P* = 0.77), the two species differed in the number of transcripts in each expression category (Table 1, χ^2^_4_ = 302.896, *P* < 0.001). *Aphaenogaster picea* induced significantly more transcripts in response to both temperature extremes (Bimodal transcripts in Table 1; χ^2^_1_ = 71.617, *P* < 0.001) than did *A. carolinensis*, which downregulated more transcripts under these conditions (Intermediate transcripts in Table 1; χ^2^_1_ = 256.329, *P* < 0.001). Consistent with the *enhanced response* hypothesis, the cool-climate *A. picea* induced 273 (~50 %) more transcripts in response to low temperatures than the warm-climate *A. carolinensis* (Low transcripts in Table 1; χ^2^_1_ = 71.227, *P* < 0.001). However, there was no difference among species in the number of transcripts upregulated at high temperatures (High transcripts in Table 1; χ^2^_1_ = 0.53, *P* = 0.47).

**Table 1:** Table of the number of thermally responsive transcripts by expression type for *A. carolinensis* and *A. picea*

	Low	Intermediate	High	Bimodal	NotResp
*A. picea*	1193	249	248	278	110
*A. carolinensis*	920	680	232	117	129

Low are transcripts with increased expression at low temperatures (<10 °C), Intermediate are transcripts with maximum expression between 10–30 °C, High are transcripts with increased expression at high temperatures (>31 °C), Bimodal are transcripts with increased expression at both low and high temperatures, while NotResp are transcripts that are not thermally responsive in one species but are in the other species

In addition, we also examined the specific patterns of shifts from one expression category to another between species. As transcripts may change expression between species due to neutral drift alone, we used the Stuart-Maxwell test of marginal homogeneity to test if the number of responsive transcripts in each expression category differed between the species when controlling for overall differences in the number of responsive transcripts. We found that the expression categories of individual transcripts between the two species were not randomly distributed (Stuart-Maxwell test of marginal homogeneity χ^2^_4_ = 319, *P* < 0.001, Additional file 2: Figure S1). Specifically, the two species differed significantly in expression pattern, which captures differences in slope as well as category, for 1553 (62 %) of the thermally responsive transcripts. Most of these shifted from one to another expression category.

**Fig. 2:**
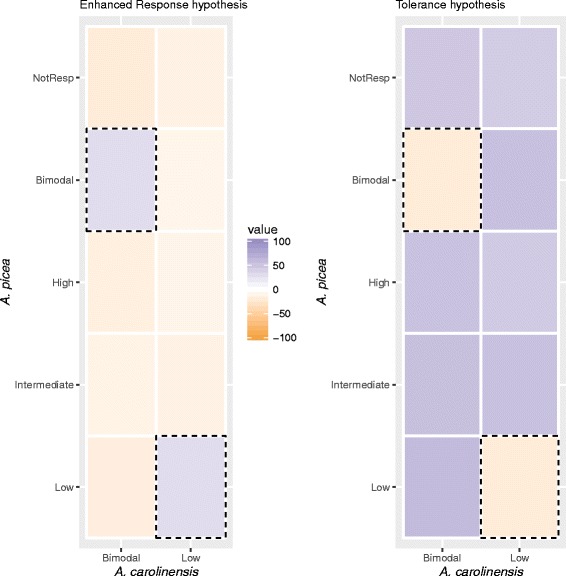
Illustrations of the expected thermal response patterns in the two species under alternative mechanistic hypotheses of temperature adaptation. Although both temperature extremes were investigated in a similar way, for simplicity only the response to low temperatures is illustrated here. Each column indicates the distribution across all response categories in *A. picea*, which has a lower CT_min_ and is therefore better adapted to low temperatures, for the set of transcripts identified as cold-induced (either High or Bimodal categories) in the species with higher CT_min_ , *A. carolinensis*, relative to the null hypothesis of equal marginal frequencies. The dashed boxes highlight cells that would indicate matched responses in the two species, and the color of each cell (blue = excess, orange = deficit) represents the deviation of the observed from expected number of transcripts. The *Enhanced Response* hypothesis (left) proposes that the increase in cold tolerance in *A. picea* is achieved by amplifying existing molecular mechanisms, and thus there should be an excess of shared response types between species. In contrast, the *Tolerance Hypothesis* (right) predicts that *A. picea* is less reliant on induced responses to confer cold-tolerance than *A. carolinensis*, leading to an excess of shifts from induction in *A. carolinensis* to the Not Responsive or down-regulation categories in *A. picea*

The *enhanced response* and *tolerance* hypotheses make opposing predictions concerning the overlap in response patterns between the two species (Fig. 2). The *enhanced response hypothesis* posits that temperature adaptation uses existing mechanisms for thermal resistance, which should result in significant overlap in response and fewer transcripts shifting expression categories than expected by chance (Fig. 2, left). In contrast, the *tolerance hypothesis* predicts that transcripts involved in active defense will become non-responsive or shift to other expression categories in the better-adapted species (Fig. 2, right). We tested these predictions by examining if the transcripts upregulated in response to the temperature extreme experienced less frequently by a species (cool temperatures for the warm-climate *A. carolinensis*, and warm temperatures for the cool-climate *A. picea*) displayed the same response profile in the species that more frequently experiences those conditions.

**Fig. 3:**
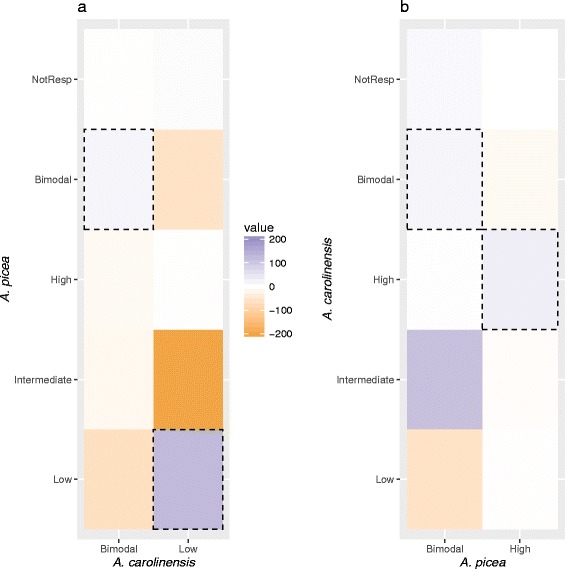
Results of analysis of thermal response patterns in the two species. The color of each cell (blue = excess, orange = deficit) represents the deviation of the observed from the expected number of transcripts based on hypothetical equivalence of the marginal frequencies. The units are number of transcripts. For each temperature extreme, the species expected to be less well adapated to that extreme is displayed on the x-axis for the two response categories corresponding to upregulation (Bimodal and Low for the low temperatures, or Bimodal and High for high temperatures). The distribution of response categories for those transcripts in the better-adapted species is arrayed along the y-axis. The dashed boxes indicate the matched responses (e.g. High - High). **a** Low temperature extreme: there is an excess of shared Low and Bimodal expression types and a bias away from all other categories in *A. picea*, consistentwith the *enhanced response* hypothesis (Fig. 1). **b** High temperature extreme: in addition to an excess of matched categories, there is an excess of High and Bimodal transcripts in A. picea that are not upregulated in *A. carolinensis* (Intermediate and Not Responsive), partially consistent with the *tolerance* hypothesis. The complete set of matched observations is shown in Additional file 1: Figure S1. Expression types are defined in Table 1

Transcripts upregulated at low temperatures in *A. carolinensis* (Low and Bimodal transcripts) were significantly biased toward this same category and away from other expression categories in *A. picea* (Fig. 3a), suggesting shared response pathways as predicted by the *enhanced response* hypothesis. In contrast, transcripts upregulated in response to high temperatures in *A. picea* (High and Bimodal) shifted expression categories in *A. carolinensis* (Fig. 3b), primarily to the Intermediate category (Fig. 3b). These transcripts are less likely to be upregulated in any context, consistent with the *tolerance hypothesis*.

### Molecular processes suggest a generalized stress response mechanism

The gene set enrichment analysis revealed a number of gene groups enriched in each expression category (Additional file 3: Table S2). Across both species, there were 9 terms enriched in the Bimodal category, including terms involved in stress response (regulation of cellular response to stress, signal transduction by p53 class mediator), cell death (apoptotic signaling pathway) and cellular organization (e.g. protein complex localization). The 6 terms enriched in the Low category suggest that proteins undergo structural (e.g. protein acylation) and organizational (single-organism organelle organization) changes to tolerate colder temperatures, possibly to maintain membrane fluidity [50]. The High category included only a single enriched GO term, “nicotinamide metabolic process”, while the Intermediate category had 5 terms including DNA packaging and metabolic process terms.

### *A. carolinensis* has greater inertia of expression change to increases in temperature than does *A. picea*

**Fig. 4:**
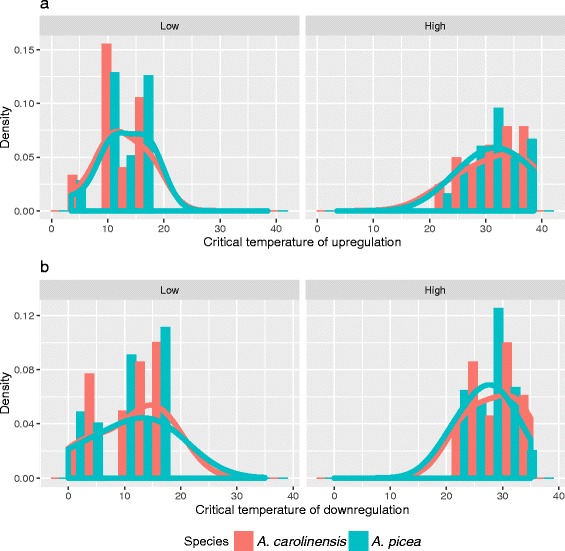
Histogram with smooth density estimate of temperature of maximum rate of change in expression for transcripts that have (**a**) increased expression at Low and High temperatures and (**b**) decreased expression at Low and High temperatures. Red bars and lines are for *A. carolinensis* while blue bars and lines are for *A. picea*

As an additional test of the *tolerance hypothesis*, we examined the critical temperature of gene induction in response to increasing and decreasing temperatures. We compared between species the mean temperatures of transcript upregulation, defined as the temperature at which the transcript showed the greatest positive change in expression. In support of the *enhanced response* but not the *tolerance hypothesis*, the temperature of induction at low temperatures was significantly higher for the cool-climate *A. picea* than for *A. carolinensis* (12.4 °C) than *A. picea* (13.1 °C; *t*_1308_ = −3.1, *P* < 0.002; Fig. 4a), though the temperature of induction did not differ between species for high temperatures (*t*_567_ = 0.8, *P* < 0.403).

Similarly, for down-regulated (Intermediate) transcripts, we compared the mean temperatures of downregulation of transcript expression between species at both high (>20 °C) and low (<20 °C) temperatures. Consistent with the *tolerance hypothesis*, *A. carolinensis* had greater inertia of gene expression in response to increasing temperatures. The temperature of downregulation for Intermediate transcripts was 28.6 °C for *A. carolinensis* compared to 27.2 for *A. picea* (*t*_294_ = 3.8, *P* < 0.001). The difference between species was not significant with decreasing temperatures (*t*_251_ = 0.5, *P* = 0.584, Fig. 4b).

### No evidence for *genetic assimilation*

**Fig. 5:**
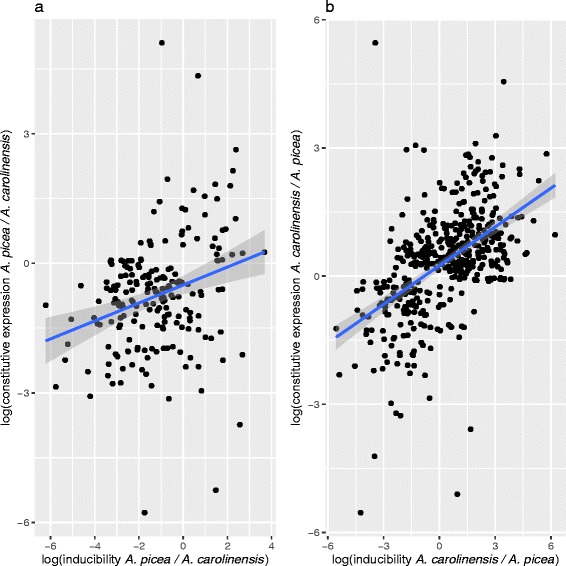
Scatterplots of log ratios of relative inducibility to relative constitutive expression, defined as expression level at the common rearing temperature (25 °C) for (**a**) High transcripts in *A. picea* (*P* < 0.001, r^2^ = 0.07) and (**b**) Low transcripts in *A. carolinensis* (*P* < 0.001, r^2^ = 0.1). Blue lines and confidence intervals are from ordinary least squares regressions

We tested the *genetic assimilation hypothesis* by comparing the log ratios of relative inducibility to relative baseline expression at the rearing temperature (25 °C). If stress-response transcripts have shifted between species from inducible to constitutive expression, there should be a negative relationship between the two. We found no evidence of such a relationship for either temperature extreme: transcripts more upregulated at high temperatures in the cool-climate *A. picea* were not expressed at higher baseline levels in the warm-climate *A. carolinensis* (Fig. 5a). Similarly, transcripts more upregulated at low temperatures in *A. carolinensis* did not show higher baseline levels in *A. picea* (Fig. 5b). In fact, for both comparisons we found a weakly positive relationship between relative inducibility and baseline expression between the two species (β_1_ = 0.31, *P* < 0.001 and (β_1_ = 0.21, *P* < 0.001). In addition, the thermally responsive transcripts in *A. carolinensis*, regardless of expression pattern, had higher baseline expression than those in *A. picea*, including those with Intermediate expression profiles in both species (Wilcoxon V = 68842, *P* < 0.001). An important exception to this pattern is the set of transcripts that had High or Bimodal expression in *A. picea* but were not thermally responsive in *A. carolinensis* (top-row of Fig. 3b). These transcripts are less likely to be upregulated in any context, consistent with the *tolerance hypothesis*.

## Discussion

The potential for many species to persist in face of climate change will depend in part upon their thermal tolerances. However, for most species little is known about how plasticity or adaptive changes in gene expression underlie thermal tolerance. By using a *reactionome* approach, we were able to quantitatively describe plasticity in transcript expression across a thermal gradient, and identify putative changes in gene expression associated with shifts in thermal tolerance between the ant species *Aphaenogaster picea* and *A. carolinensis*. We found non-linear patterns of gene expression changes in response to temperature, with both quantitative and qualitative differences between species, consistent with different mechanisms of thermal adaptation to low and high temperature extremes.

Under the *enhanced response* hypothesis, stress-adapted species are hypothesized to induce a stronger and earlier response to extreme conditions. We found evidence for this hypothesis at low temperatures: although the lower thermal limit for *A. picea* is substantially lower than *A. carolinensis*, *A. picea* upregulated responsive transcripts at slightly less extreme temperatures (Fig. 4a). Moreover, the transcripts upregulated in *A. picea* included about about half (55 %) those upregulated in *A. carolinensis* as well as an additional set of 261 transcripts (Table 1), enriched for metabolism, organization and translation processes (Additional file 3: Table S2). Two non-mutually exclusive hypotheses may explain this pattern. First, surviving prolonged low temperatures, such as would be experienced during overwintering, generally requires advance production of specialized cryoprotectants [43] and a suite of preparatory physiological modifications [51]. The northern species *A. picea* may induce a greater response to survive the longer winter period. Alternatively, the response to low temperatures may reflect countergradient expression to counteract reduction in enzyme efficiency, and maintain activity as temperature declines [41]. This requirement may be under stronger selection in *A. picea* given the shorter growing season that would necessitate foraging under a broader range of temperatures.

In contrast to cold tolerance, the enhanced upper thermal limit in *A. carolinensis* is best explained by the *tolerance* hypothesis. High temperatures were associated with significantly fewer upregulated transcripts in *A. carolinensis* (Table 1), and a large proportion (25 %) of the transcripts upregulated at high temperatures in *A. picea* were either downregulated or expressed at negligible levels overall in *A. carolinensis*. These results suggest that mechanisms other than the heat shock response are acting to maintain protein stability in face of temperature increases. Such mechanisms may include novel constitutive defenses [19, 21, 22], enhanced proteome stability [52] or behavioral quiescence [5] to tolerate thermal stress. These differences are in line with expectations that *A. carolinensis*, with a growing season over twice the length of its northern congener, may be better able to afford to restrain from foraging in suboptimal conditions. Indeed, quiescence under stressful conditions by the red harvester ant *Pogonomyrmex barbatus* has been shown to increase colony fitness [42].

The one hypothesis that did not receive support was the *genetic assimilation hypothesis* (Fig. 5), which predicts that exposure to more frequent stressors will select for a shift from inducible to constitutive expression of stress-response transcripts. This contrasts with other recent studies on adaptation in field populations to thermal stress [21]. However, in a short-term selection experiment for heat tolerance, Sikkink et al. [46] also found no evidence for genetic assimilation at the expression level after ten generations of selection for heat tolerance in *Caenorhabditis remanei*, even though there was a substantial increase in heat tolerance. Both the genetic assimilation and tolerance routes to increasing thermal limits are functionally similar in that they emphasize damage prevention rather than repair. Whether a particular taxon evolves one strategy over another may be related to availability of alternative mechanisms as well as the the intensity, frequency and duration of temperature stress in a given environment.

Given the differences in the patterns of thermal responsiveness between species (Fig. 3), it is worth noting a number of similarities. In both species, there were 2 -- 3 times more transcripts upregulated at low than high temperatures (Table 1). The degree of upregulation at low temperatures is surprising given previous studies [53, 54] that found little transcriptional activity at low temperatures. However, these studies exposed organisms to a few extreme (−10 -- 0 °C) temperatures. At these extremes, we also found few upregulated transcripts (Fig. 3a), whereas the peak of low-temperature transcriptional activation occurred near 10 °C (Fig. 4). A potential explanation for this pattern is that increased gene expression functions to support elevated metabolism at moderately cold temperatures, as suggested by the metabolic cold adaptation hypothesis [55]. The observation that more transcripts were upregulated at low than high temperatures could also be due to stronger selection on upper than lower thermal limits, thereby reducing both genetic variation and gene expression plasticity at high temperatures [4, 56]. This explanation is consistent with the observation in *Aphaenogaster rudis* [57] and other ectotherms [10, 58] that critical maximum temperatures vary less among taxa than do critical minimum temperatures.

Critical maximum and minimum temperatures are hypothesized to be genetically correlated [10, 58], but this was not evident in terms of gene expression in this study. Only ~10 % of transcripts upregulated in response to temperature were bimodal, and for both activation and down-regulation, thresholds differed between species at only one temperature extreme (Fig. 4). This suggests that species do not face a fundamental trade-off between these two limits and may be able to shift upper and lower thermal limits independently to match requirements of more seasonally variable environments.

A major contribution of this study is the construction of a reactionome for gene expression data. Similar approaches have been used in other species [59, 60], but to our knowledge, none have applied a regression approach to identify a complete list of responsive transcripts across an environmental gradient. This approach revealed quantitative patterns of temperature response not captured in categorical comparisons. For example, the degree of upregulation at cool (~10 °C) but not extreme cold temperatures was missed in previous studies that focused on extreme cold limits, as discussed above. Further, a number of issues have hampered RNA-seq studies to date. Namely, lists of differentially expressed transcripts are prone to false positives [61], depend on the genetic background of the organism [62] and are prone to “storytelling” interpretations [63]. Our findings are robust to these issues as we focus on the average change in the shape of the reaction norms across many hundreds of responsive transcripts in each species. Although we use gene ontology information to interpret our results, the key findings about differential plasticity of expression between species do not depend on functional annotation.

Moreover, by characterizing responses across thousands of transcripts, the reactionome approach can help to distinguish selection from neutral drift in gene expression [64–66]. Although we cannot rule out drift as a source of variation for individual transcripts, we would not expect to see systematic differences in expression type categories or critical temperature thresholds as we do here (Fig. 3, Additional file 2: Figure S1). Thus, our method provides an example of how focusing on transcriptome-wide changes in gene expression—as opposed to identifying lists of differentially-expressed transcripts—can provide meaningful insight on the process of evolution. It should be noted, however, that although including non-linear relationships between expression and temperature captured a significantly larger range of biologically-relevant responses, it also led to a substantial increase in false positives. Empirical estimation of these rates via randomization tests, combined with robust sampling designs, can help to minimize this bias and focus results on biologically-meaningful gene sets.

A number of caveats do apply to our work. First, species may differ in gene expression along axes which we have not measured here, especially temporal patterns of gene expression [25], which could be studied in further work. Second, the *de novo* transcriptome assembly is highly fragmented, given that all sequenced ant genomes to date have only about 18,000 genes [67]. Although we took steps to remove contaminants and redundant transcripts, some likely remain, in addition to partially assembled transcripts. A genome assembly, in progress, will help to reduce fragmentation. Third, the quality of the annotation for a non-model system such as *Aphaenogaster* is not as good as it would be for model arthropods such as *Drosophila* and *Apis*. Finally, the mapping of changes in gene expression to organismal fitness is far from direct [68], and large differences in patterns of gene expression may have only small effects on fitness. In particular, functional protein levels cannot be expected to be fully linked to mRNA abundance due to post-transcriptional modification, regulation, mRNA fluctuations and protein stability [68].

Our results are congruent with evidence from other systems [21] that thermally-stressful habitats select for investment in tolerance, whereas organisms from less stressful environments rely on plastically-induced resistance. Although the heat-shock response is one of the most conserved across living organisms [39], it is energetically expensive, particularly under chronic stress conditions [69]. Under such circumstances, it may be advantageous to proactively prevent thermal damage even at the cost of reduced metabolic efficiency, either by maintaining a higher constitutive level of chaperone proteins [11] or by increasing the thermal stability of proteins at the expense of catalytic activity [70]. Thus, although in the short term increasing temperature stress leads to a quantitatively stronger induced response, adapting to such stress over evolutionary time appears to require a qualitative shift in mechanism of resistance that can alter not only the magnitude, but the sign of gene expression change in response to temperature. Whether such a shift would be possible in the compressed time frame of projected climate change, particularly for long-lived organisms such as ants, is likely to be critical in determining the capacity of populations to adapt to more frequent and long-lasting stressors.

## Conclusions

In this work, we have brought reaction norms to the genomic era by characterizing the thermal reactionomes of two temperate ant species, *Aphaenogaster picea* and *A. carolinensis*. At least 2 % of their transcriptomes are thermally responsive. Our results indicate that these two ant species have different responses to thermal extremes. *A. picea* responds by increasing expression of transcripts related to metabolism, stress response and other protective molecules, whereas *A. carolinensis* decreases expression of transcripts related to metabolism and likely relies on other mechanisms for thermal tolerance. The thermal reactionomes of these two species provide key insights into the genetic basis of thermal tolerance, and a resource for the future study of ecological adaptation in ant species. Finally, the reactionome itself illustrates a new direction for characterizing acclimation and adaptation in a changing climate.

## Methods

### Samples

Ants of the genus *Aphaenogaster* are some of the most abundant in eastern North America [71], and species as well as populations within species differ in critical maximum and minimum temperatures [57]. Temperature is a potentially strong selective force for ground-nesting ant populations, which must tolerate seasonally freezing winters and hot summers. On shorter time scales, individual workers can experience extreme thermal environments when they leave the thermally buffered ant nest to forage for food [32].

In fall 2012, we collected a single colony of *Aphaenogaster picea* from Molly Bog, Vermont (University of Vermont Natural Areas; 44.508° N, −72.702° W) and a single colony of *Aphaenogaster carolinensis*, part of the *A. rudis* species complex [26], from Durham, North Carolina (36.037° N −78.874° W). These sites are centrally located within each species’ geographic range. Species identity was confirmed with morphological characters (Bernice DeMarco, Michigan State University). Colonies of both species were maintained in common conditions at 25 °C for 6 months prior to experimentation. Due to colony size limitations, we were unable to determine the critical thermal limits of these particular colonies. In summer 2013 we collected additional colonies of *Aphaenogaster* from Molly Bog, VT and North Carolina (Duke Forest, 36.036° N, 79.077° W). We tested the upper and lower critical thermal limits for five ants from each of these colonies using a ramp of 1 °C per minute, starting at 30 °C, and recorded the temperature at which the ants were no longer able to right themselves, following the protocol of Warren & Chick [57].

### Common garden design

Ideally, genetically-based variation in gene expression profiles would be identified by comparing individuals completely reared under common-garden conditions to eliminate environmental variation experienced either as adults or during development. However, *Aphaenogaster* colonies are long-lived, cannot be bred under laboratory conditions, and do not achieve complete turnover of the workforce for at least a year or longer. Thus, as is commonly done with other long-lived organisms [21, 65], we exposed both colonies to common-garden rearing conditions for six months to fully acclimate adult workers to common temperatures. Over this time, roughly 1–2 cohorts of new workers are expected to join each colony (~1/3 of the total), such that the workers sampled for thermal traits and gene expression are likely to have included a mix of adult-acclimated and fully lab-reared individuals.

Unlike ANOVA-based experimental designs, which derive statistical power from replication within each experimental treatment level, regression designs have greater power when sampling additional values across the range of the continuous predictor variable [72]. Ideally, the treatments should be replicated at each level of the predictor variable [73]. However, even with no replication, the regression design is still more powerful than an ANOVA design with comparable replication, and provides an unbiased estimator of the slope [72]. For these reasons, we focused our sequencing efforts on maximizing the number of temperatures at which the transcriptome was profiled, rather than on replication at each temperature.

To limit differences in gene expression not related to the experimental treatment, on 12 different days we haphazardly collected three ants from each 2012 colony at the same time of day to minimize variation due to circadian oscillations. We measured response to temperature with a one-hour static temperature application, which is ecologically relevant for workers that leave the thermally-buffered nest and are immediately exposed to ambient temperatures while foraging [71]. Each day, the ants were placed in glass tubes immersed in a water bath maintained at one of 12 randomly-assigned temperatures (0° to 38.5 °C, in 3.5° increments) for one hour. The minimum and maximum temperatures were selected based on previous work showing that these temperatures are close to the critical minimum (~0 °C) and maximum (~43 °C) temperatures for *Aphaenogaster* [57], and these treatments did not cause mortality. At the end of the hour, the ants were flash frozen in liquid nitrogen and stored at −80 °C. Thus, our reactionome characterized early, but not late, responding genes. We extracted mRNA by homogenizing the three pooled ants in 500 uL of RNAzol buffer with zirconium silicate beads in a Bullet Blender (Next Advance; Averill Park, NY), followed by RNAzol extraction (Molecular Research Center Inc; Cincinnati, OH) and then an RNeasy micro extraction (Qiagen Inc; Valencia, CA) following the manufacturer’s instructions.

### Sequencing, assembly and annotation

For each species, the 12 samples were barcoded and sequenced in a single lane of 2 × 100 bp paired-end reads on an Illumina HiSeq 1500 yielding 200 and 160 million reads for the *A. picea* and *A. carolinensis* samples respectively. Reads were filtered to remove Illumina adapter sequences and low quality bases using the program Trimmomatic [74].

We assembled the sequenced reads into the full set of mRNA transcripts, the transcriptome, for the combined data set from both species using the Trinity *de novo* transcriptome assembly program [75]. *De novo* transcriptome assembly is prone to falsely identifying alternative transcripts and identifying inaccurate transcripts that are chimeric (e.g. regions of two separate transcripts that assemble into a false, or chimeric, third transcript) [76]. We removed potentially false transcripts by first running the program CAP3 [77] to cluster sequences with greater than 90 % similarity and merge transcripts with overlaps longer than 100 bp and 98 % similar in length. Second, we ran the program uclust which clusters sequences completely contained within longer sequences at greater than 90 % similarity (see Additional file 4). We used liberal values (90 % similarity) to merge orthologous transcripts in the two species that may not have assembled together in the initial *de novo* transcriptome assembly. To identify contaminant sequences, we screened our full transcriptome using the program DeconSeq [78] with the provided bacteria, virus, archaen and human databases of contaminants.

The Trinity *de novo* transcriptome assembly for both species assembled together included 126,172 transcripts with a total length of 100 million bp. Filtering to remove redundant or chimeric reads resulted in an assembly with 105,536 transcripts. The total length was 63 million bp with an N_50_ length of 895 bp and a mean transcript size of 593 bp. Of the 105,536 filtered transcripts, 55,432 had hits to the NCBI-nr database. Of these, 38,711 transcripts mapped to GO terms, 1659 transcripts were identified to an enzyme and 18,935 transcripts mapped to a domain with >50 % coverage. We removed 5675 transcripts identified as known contaminants, leaving 99,861 clean transcripts.

We assessed the quality of the transcriptome using the BUSCO program [47] available from (http://busco.ezlab.org/). BUSCO asseses transcriptome completeness by measuring the number of near-universal single-copy orthologs using the Arthropod database from OrthoDB.

To determine the putative function of the transcripts, we used functional annotation of the transcriptome assembly using the web-based tool FastAnnotator [79] which annotates and classifies transcripts by Gene Ontology (GO) term assignment, enzyme identification and domain identification.

### Identification of thermally-responsive transcripts

We quantified expression of each transcript using the program Sailfish [48] and used the bias-corrected transcripts per million (TPM) [80] as our measure of transcript expression. We included the contaminant transcripts identified by DeconSeq at the quantification stage to avoid incorrectly assigning reads to other transcripts, but removed these from further analyses. Because preliminary examination of the data (Additional file 4) indicated that the 7 °C samples may have been mis-labeled, we omitted these data from the analysis. The expression values were highly correlated between species at each temperature treatment (r^2^ > 0.98) indicating that assembling the transcriptome with data from both species was justified (Additional file 3).

To identify transcripts that had significant changes in expression across the thermal gradient, we fit to each transcript an ordinary least-squares polynomial regression model

$$ \begin{array}{l} \log \left(\mathrm{T}\mathrm{P}\mathrm{M}+1\right)={\upbeta}_0+{\upbeta}_1\left(\mathrm{species}\right)+{\upbeta}_2\left(\mathrm{temperature}\right)+{\upbeta}_3\left({\mathrm{temperature}}^2\right)+\\ {}{\upbeta}_4\left(\mathrm{species}*\mathrm{temperature}\right)+{\upbeta}_5\left({\mathrm{species}*\mathrm{temperature}}^2\right)+\upepsilon \end{array} $$

Temperature and species were both fixed effects, with a quadratic term included for temperature. We used log(TPM + 1) as the response to control for skew in the expression data. For a continuous predictor such as temperature, this regression approach is preferred to an ANOVA approach as it can reveal non-linear responses such as hump-shaped or threshold effects [72]. This method is robust to over-dispersion because we expect errors in the read count distribution [81] to be independent with respect to temperature.

To evaluate the statistical significance of the patterns, we computed parametric *P*-values for each model and adjusted these *P*-values using the False Discovery Rate (FDR) approach of Benjamini and Hochberg [82]. As a more stringent filter for false positives, we then randomly re-assigned each transcript within a species to a different temperature, fit the polynomial models as above, and again calculated *P*-values and FDR. Ideally, these randomized data sets should not yield any significant associations. We repeated this resampling approach 100 times, and used the 95th quantile of false significant transcripts as the null expectation for retaining transcripts from the true data.

Of these overall significant transcripts, we identified thermally-responsive transcripts as the subset that had significant β_2_(temp), β_3_(temp^2^),β_4_(species * temp) or β_5_(species * temp) terms after step-wise model selection by AIC. For each thermally-responsive transcript, we predicted expression levels using the final linear model for each species across the tested thermal range. We used the predicted transcript expression levels to partition transcripts for each species into five expression categories: High transcripts had greatest expression at temperatures > 31 °C, Low transcripts had greatest expression at temperatures < 10 °C, Intermediate transcripts had greatest expression between 10 to 30 °C, Bimodal transcripts had increased expression at both high and low temperatures, while NotResp transcripts were those that were not thermally responsive in the focal species but did respond in the other. For the Bimodal group, we required that expression at both low and high temperatures was at least one standard deviation greater than the expression at the rearing temperature of 25 °C. Because expression category was defined by the temperature of maximal expression, both Low and High categories were biased toward transcripts up-regulated at that temperature extreme, but also likely included some transcripts down-regulated at the opposing extreme. The two categories which could unambiguously distinguish up- from down-regulation are *Bimodal* (up at both extremes) and *Intermediate* (down at both extremes).

### Statistical analyses

We used *χ*^2^ tests to determine if the total number of responsive transcripts, and the number of transcripts in each expression category differed between species. To evaluate if shifts from one expression category to another between the two species were randomly distributed, we used the Stuart-Maxwell test of marginal homogeneity from the coin package [83] in R [84] which tests if the row and column marginal proportions are in equity.

To test whether the temperature at which thermally-responsive transcripts were activated differs between species, we identified the temperature at which there was the greatest change in expression for each transcript in each species, using only the transcripts with a significant species x temperature interaction. For upregulated transcripts, we grouped the High transcripts along with the high temperature end of the Bimodal transcripts, and did the same for Low transcripts. We then performed a *t*-test to determine if the mean temperature of transcript activation differed between the two species for each group. For downregulated transcripts (i.e. Intermediate), we identified the greatest change in expression for each transcript in response to both increasing (>20 °C) and decreasing (<20 °C) temperatures, and used a *t*-test to compare the mean temperature of down-regulation between species.

To test for a tradeoff between induciblity and constitutive baseline expression between species, we fit ordinary least squares regressions with the log ratio of relative constitutive expression as the response variable and the log ratio of relative inducibility as the predictor variable for High transcripts in *A. picea* and for Low transcripts in *A. carolinensis*. Constitutive expression was defined as predicted expression at 25 °C, whereas inducibility of each transcript was defined as *((maximum TPM - minimum TPM)/minimum TPM) x 100*. In addition, we used a Mann–Whitney test to compare the baseline constitutive expression between species for all responsive transcripts.

### Gene set enrichment analysis

To describe the molecular processes involved in thermal adaptation, we performed gene set enrichment analysis (GSEA) using the parentChild algorithm [85] from the package topGO [86] in R [84]. Briefly, this approach identifies GO terms that are overrepresented in the significant transcripts relative to all GO terms in the transcriptome, after accounting for dependencies among the GO terms.

All analyses were performed with R 3.2 [84] and are fully reproducible (Additional file 4).

### Availability of supporting data

The reproducible and version-controlled scripts underlying the analysis are available at http://dx.doi.org/10.5281/zenodo.46416.

The Illumina short-read sequence data supporting the results of this article are available in the NCBI Short Read Archive BioProject repository, PRJNA260626 http://www.ncbi.nlm.nih.gov/bioproject/PRJNA260626/.

The Trinity transcriptome assembly, FastAnnotator annotation file and Sailfish gene expression quantification files supporting the results of this article are available from the LTER data portal, datasets hf113-38, hf113-41, and hf113-42 (http://dx.doi.org/10.6073/pasta/05ea6464df30efa2f1e2c7439366bf47).

## Additional files

Additional file 1: Table S1. Annotation, *P*-value, r^2^ , adjusted *P*-value, and expression type for the thermally-responsive transcripts in each species. (CSV 436 kb) Additional file 2: Figure S1. Deviations from expected numbers of transcripts in matched observations of transcript expression type between species (*A. carolinensis* on rows, *A. picea* on columns). The color of each cell represents the deviation of the observed from the expected number of transcripts based on hypothetical equivalence of the marginal frequencies (blue = excess, orange = deficit). The expression types are Low transcripts that had greatest expression temperatures < 10 °C, Intermediate transcripts with greatest expression between 10 and 30 °C, High transcripts that had greatest expression at temperatures > 31°, Bimodal transcripts with increased expression at both high and low temperatures, and Not Responsive transcripts that were not thermally responsive in that species. (PNG 19 kb) Additional file 3: Table S2. Results of the gene set enrichment analysis, showing the enriched gene ontology terms for each species in each thermal response category. (CSV 2 kb) Additional file 4: Technical Report containing full details of the analysis. (PDF 970 kb)
