# Supplementary figures and images for: Thermal reactionomes reveal divergent responses to thermal extremes in warm and cool-climate ant species
Source: BMC Genomics. 2016 Mar 2;17:171. doi: 10.1186/s12864-016-2466-z (PMC4776372; doi:10.1186/s12864-016-2466-z)

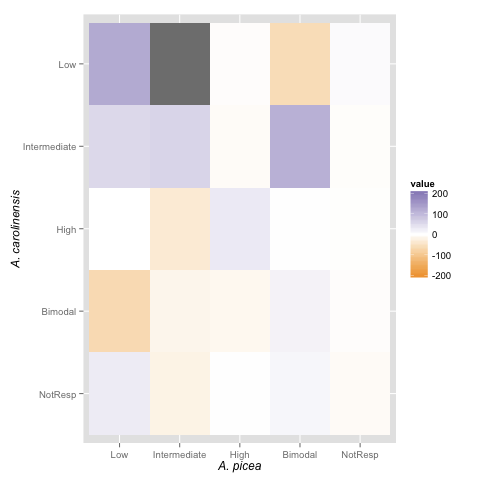

Supplement: Additional file 2: — Figure S1. Deviations from expected numbers of transcripts in matched observations of transcript expression type between species (A. carolinensis on rows, A. picea on columns). The color of each cell represents the deviation of the observed from the expected number of transcripts based on hypothetical equivalence of the marginal frequencies (blue = excess, orange = deficit). The expression types are Low transcripts that had greatest expression temperatures < 10 °C, Intermediate transcripts with greatest expression between 10 and 30 °C, High transcripts that had greatest expression at temperatures > 31°, Bimodal transcripts with increased expression at both high and low temperatures, and Not Responsive transcripts that were not thermally responsive in that species. (PNG 19 kb) [file 12864_2016_2466_MOESM2_ESM.png]
