# Supplementary material for: Thermal reactionomes reveal divergent responses to thermal extremes in warm and cool-climate ant species
Source: BMC Genomics. 2016 Mar 2;17:171. doi: 10.1186/s12864-016-2466-z (PMC4776372; doi:10.1186/s12864-016-2466-z)
Supplement: Additional file 4: — Technical Report containing full details of the analysis. (PDF 970 kb) [file 12864_2016_2466_MOESM4_ESM.pdf]

Technical Report for Thermal reactionomes reveal divergent responses to thermal extremes in warm and cool-climate ant species

Author: John Stanton-Geddes

29 September, 2015

Technical Report No. 3

Department of Biology

University of Vermont

Summary

In this technical report, which accompanies the manuscript **Thermal reactionomes reveal divergent responses to thermal extremes in warm and cool-climate ant species**, we:

1. Describe the *de novo* assembly of the transcriptome for two ant species within the *Aphaenogaster rudis-picea-fulva* species complex (Lubertazzi, 2012).
2. Measure the reaction norm across a thermal gradient (0°C to 38.5°C) for all transcripts and characterize the *thermal reactionome* as the set of transcripts that are thermally-responsive.
3. Quantitatively test three mechanistic hypotheses of thermal adapation:.

- The *Enhanced response* hypothesis proposes that species extend their thermal limits through a stronger induced response to provide greater protection from more frequently encountered stressors.
- The *Tolerance hypothesis* proposes that existing inducible stress responses become insufficient or prohibitively costly as environmental stressors increase in frequency.
- The *Genetic assimilation hypothesis* proposes that exposure to more extreme stressors selects for a shift from inducible to constitutive expression of stress-response genes.

We quantified the relative importance of each of these mechanisms to thermal adaptation in these two species by evalutating differences in the reactionomes between species. We used gene set enrichment analysis to provide additional insight on the cellular processes underlying the transcriptome-wide changes in expression.

Data

The raw Illumina fastq files are available from the NCBI short read archive [link tbd]. The assembled transcriptome, annotation and expression values are available from the Harvard Forest Archives, HF-113 [http://harvardforest.fas.harvard.edu/data-archive] datasets hf113-36, hf113-40 and hf113-41. These processed files are used for this analysis due to the computational demands of re-generating the files, but the exact commands for each of these steps are documented below.

Sample description

Two ant colonies were used for the transcriptome sequencing. The first, designated *A22*, was collected at Molly Bog, Vermont in August 2012 by Nick Gotelli and Andrew Nguyen. The second colony, designated *Ar*, was collected by Lauren Nichols in Raleigh, North Carolina. These colonies were maintained in the lab for 6 months prior to sample collection. Bernice DeMarco (Michigan State University) identified colony *A22* as *A. picea* and *Ar* as *A. carolinensis*.

For each colony, three ants were exposed to one of 12 temperature treatments, every 3.5°C ranging from 0°C to 38.5°C, for one hour in glass tubes in a water bath. The ants were flash frozen and stored at -80C until RNA was extracted using a two step extraction; RNAzol RT (Molecular Research Center, Inc) followed by an RNeasy Micro column (Qiagen). Samples from each colony were pooled and sequenced in separate lanes on a 100bp paired-end run of an Illumina HiSeq at the University of Minnesota Genomics Center, yielding 20e6 and 16e6 reads for the A22 and Ar samples, respectively.

Transcriptome assembly

The Illumina reads were filtered using the program Trimmomatic (Lohse, Bolger, Nagel, Fernie, Lunn, Stitt, and Usadel, 2012) to remove Ilumina adapter sequences, trim bases with PHRED quality scores less than 15 in a 4 bp sliding window and remove final trimmed sequences with length less than 36 bp. The code used was

```
java -jar trimmomatic-0.30.jar PE -threads 40 -phred33 -trimlog trimmomatic.log \\
sample.R1.fastq sample.R2.fastq sample.R1.trimmed.paired.fastq sample.R1.trimmed \\
.unpaired.fastq sample.R2.trimmed.paired.fastq sample.R2.trimmed.unpaired.fastq \\
ILLUMINACLIP:TruSeq3-PE.fa:2:30:10 LEADING:3 TRAILING:3 SLIDINGWINDOW:4:15 MINLEN:36
```

where "sample" was replaced by each sample. This filtering yielded 339,845,787 properly paired reads and 16,860,885 unpaired reads.

Properly paired and unpaired reads passing the Trimmomatic filter were combined and used in de novo transcriptome assembly using the program Trinity (Grabherr, Haas, Yassour, Levin, Thompson, Amit, Adiconis, Fan, Raychowdhury, Zeng, Chen, Mauceli, Hacohen, Gnirke, Rhind, di Palma, Birren, Nusbaum, Lindblad-Toh, Friedman, and Regev, 2011). Unpaired reads resulting from Trimmomatic were concatenated to the set of left (R1) reads per Trinity usage documentation. Assembly on a single 40 CPU machine with 1TB of memory required about 20 hours of compute time.

```
Trinity.pl --seqType=fq --JM=90G -left=all.R1.trimmed.fastq -right=all.R2.trimmed.fastq \\
--output=trinity --CPU=40 --inchworm_cpu=40 --bflyCPU=5
```

This assembly contained 100,381 unique components (~genes) in 126,172 total transcripts (Table 1).

As we were assembling two divergent colonies into a single transcriptome, we suspected that this assembly would be susceptible to known problems of errors during assembly (e.g. chimeric transcripts that are fusions of two transcripts) and redundancy (Yang and Smith, 2013). To account for this, we performed two post-assembly processing steps.

First, we ran the program cap3 (Huang, 1999) setting the maximum gap length and band expansion size to 50 `-f 50 -a 50`, no end clipping as the reads were already filtered `k 0`, requiring 90% identity for assembly, and a minimum overlap length of 100 bp `-o 100`. The percent identity threshold of 90% was chosen to liberally collapse orthologous contigs from the two colonies that may have been assembled separately.

```
cap3 Trinity.fasta -f 50 -a 50 -k 0 -p 90 -o 100 > Trinity_cap3.out
```

The output of `cap3` gives assembled "contigs" and unassembled "singlets" that were concatenated into a single file.

```
# check the number of contigs clustered
grep -c "Contig" Trinity.fasta.cap.contigs
grep -c "comp" Trinity.fasta.cap.singlets
# compare to contigs from Trinity output
grep -c "comp" Trinity.fasta
```

```
# Combine contigs and singlets from CAP3
cat Trinity.fasta.cap.contigs Trinity.fasta.cap.singlets > Trinity_cap3.fasta
```

The output file "Trinity.fasta.cap.info" gives specific information on which contigs were collapsed.

Subsequent to running `cap3`, we ran uclust to cluster sequences completely contained within longer sequences, again specificing a 90% identity cutoff for clustering.

```
# sort
uclust --sort Trinity_cap3.fasta --output Trinity_cap3_sorted.fasta
# cluster by 90% similarity threshold
uclust --input Trinity_cap3_sorted.fasta --uc Trinity_cap3_uclust.out --id 0.90
# convert uclust to fasta format
uclust --uc2fasta Trinity_cap3_uclust.out --input Trinity_cap3_uclust.fasta
```

These post-processing step removed 16% of the initial reads (Table 1).

	Total contigs	Total length	Median contig size
**Trinity**	126,172	100,389,539	358
**Reduced**	105,536	62,648,997	320

Table 1: Statistics for Trinity and cap3+uclust reduced transcriptome assemblies (continued below)

	Mean contig size	N50 contig	N50 Length
**Trinity**	795	16,201	1,631
**Reduced**	593	15,491	895

To remove contigs that are likely contaminants from bacterial, archael, virus or human sources, we used the program DeconSeq r citep("10.1371/journal.pone.0017288"). We downloaded the bacteria, virus, archae and human databases of contaminants, modified the `DeconSeqConfig.pm` file as described here to point to the databases, and ran DeconSeq specifiying 95% identity over 50% the length of contig

```
deconseq.pl -c 50 -i 95 -f Trinity_cap3_uclust.fasta -d Trinity_cap3_uclust \\
-dbs hsref,bast,vir,arch
```

This resulted in removing 5,675 contigs as contaminants, leaving 99,861 "clean" contigs. We spot-checked the contaminants by BLAST and confirmed that they matched bacteria, human or viral sources by greater than 95%. For expression quantification, we use the full assembly to ensure that "contaminant" reads are assigned to the contaminants. After quantification, these transcripts will then be removed from further analyses.

Running Trinity and subsequent programs is time and memory-intensive so the final assembly is downloaded and used for all further analyses. The downloaded archive contains the "clean" and "contaminant" sequences after filtering with DeconSeq.

```
# download filtered Trinity assembly, uncompress and move
```

```
wget http://harvardforest.fas.harvard.edu/data/p11/hf113/hf113-40-ap-trans.tar

# move and extract
mkdir -p results/
mkdir -p results/trinity-full/
mv hf113-40-ap-trans.tar results/trinity-full/.
tar -xvf hf113-40-ap-trans.tar
```

To examine the species distribution of BLAST hits in the transcriptome assembly, I used the program Krona r citep("doi:10.1186/1471-2105-12-385") which provides a taxonomic profile of all transcripts by BLAST annotation to species.

The interactive visualization is available here.

Transcriptome annotation

Annotation was performed by uploading the reduced assembly "Trinity_cap3_uclust.fasta" to the web-based annotation program FastAnnotator.

Results were available as job ID 13894410176993.

This annotation file can be downloaded from the Harvard Forest archive, or read directly into R.

```
# URL for annotation file
annotation.URL <- getURL("http://johnstantongeddes.org/assets/files/ApTranscriptome/ApTranscriptome_Ann

# load
annotation.file <- read.csv(textConnection(annotation.URL), header = TRUE, sep = "\t", stringsAsFactors
str(annotation.file)
```

```
## 'data.frame':    105536 obs. of  12 variables:
##  $ Sequence.Name        : chr  "0|*|Contig6267" "1|*|comp150820_c2_seq6" "2|*|Contig6262" "3|*|comp1
##  $ sequence.length      : int  9990 9944 9711 9639 9558 9436 9410 9396 9103 9030 ...
##  $ best.hit.to.nr       : chr  "gi|110756860|ref|XP_392375.3| PREDICTED: hypothetical protein LOC408
##  $ hit.length           : chr  "598" "1777" "511" "1077" ...
##  $ E.value              : chr  "2.1e-265" "0.0" "1.29e-246" "0.0" ...
##  $ Bit.score            : chr  "904.618736" "3455.802741" "842.252472" "2272.638439" ...
##  $ GO.Biological.Process: chr  "GO:0035335 peptidyl-tyrosine dephosphorylation | GO:0000188 inactiva
##  $ GO.Cellular.Component: chr  "-" "-" "-" "GO:0005634 nucleus" ...
##  $ GO.Molecular.Function: chr  "GO:0017017 MAP kinase tyrosine/serine/threonine phosphatase activity
##  $ Enzyme               : chr  "3.1.3.16  | 3.1.3.48 " "-" "3.1.3.16  | 3.1.3.48 " "-" ...
##  $ Domain               : chr  "pfam00782 DSPc | pfam00581 Rhodanese" "pfam02181 FH2 | pfam00067 p45
##  $ annotation.type      : chr  "GO & Enzyme & Domain" "GO & Domain" "GO & Enzyme & Domain" "GO & Doma
```

```
# Convert to data.table
annotation.table <- data.table(annotation.file)
str(annotation.table)
```

```
## Classes 'data.table' and 'data.frame':   105536 obs. of  12 variables:
##  $ Sequence.Name        : chr  "0|*|Contig6267" "1|*|comp150820_c2_seq6" "2|*|Contig6262" "3|*|comp1
##  $ sequence.length      : int  9990 9944 9711 9639 9558 9436 9410 9396 9103 9030 ...
##  $ best.hit.to.nr       : chr  "gi|110756860|ref|XP_392375.3| PREDICTED: hypothetical protein LOC408
##  $ hit.length           : chr  "598" "1777" "511" "1077" ...
```

```
##  $ E.value              : chr  "2.1e-265" "0.0" "1.29e-246" "0.0" ...
##  $ Bit.score            : chr  "904.618736" "3455.802741" "842.252472" "2272.638439" ...
##  $ GO.Biological.Process: chr  "GO:0035335 peptidyl-tyrosine dephosphorylation | GO:0000188 inactiva
##  $ GO.Cellular.Component: chr  "-" "-" "-" "GO:0005634 nucleus" ...
##  $ GO.Molecular.Function: chr  "GO:0017017 MAP kinase tyrosine/serine/threonine phosphatase activity
##  $ Enzyme               : chr  "3.1.3.16  | 3.1.3.48 " "-" "3.1.3.16  | 3.1.3.48 " "-" ...
##  $ Domain               : chr  "pfam00782 DSPc | pfam00581 Rhodanese" "pfam02181 FH2 | pfam00067 p45
##  $ annotation.type      : chr  "GO & Enzyme & Domain" "GO & Domain" "GO & Enzyme & Domain" "GO & Dom
##  - attr(*, ".internal.selfref")=<externalptr>
```

Identification of thermally-responsive genes

Gene expression

I quantified gene expression using sailfish. To run this program, first make sure that PATHs to the software libraries are set up correctly as described on the sailfish website.

An index of the assembly is built with the command:

```
sailfish index -t results/trinity-full/Trinity_cap3_uclust.fasta -o results/trinity-full/sailfish-index
```

Once this is done, expression is quantified for the Trimmomatic filtered reads from each species-treatment sample separately. Note that for each sample, there are four filtered read files:

- paired.left.fastq
- paired.right.fastq
- unpaired.left.fastq
- unpaired.right.fastq

```
# make a directory for the expression values
mkdir -p results/trinity-full/sailfish-expression-Trinity-cap3-uclust
# change to "trinity-full" directory
cd results/trinity-full
# for each sample, run the following command
sailfish quant -i sailfish-index-Trinity-cap3-uclust -o sailfish-expression-Trinity-cap3-uclust/A22-0 -
```

While it is possible to separately specify the paired-end and orphaned single-end reads in Sailfish v0.6.3, the results are exactly the same as if they are all entered as SE.

These files are downloaded for convenience:

```
# download gene expression quantification
wget http://harvardforest.fas.harvard.edu/data/p11/hf113/hf113-41-ap-gene-exp-quant.tgz
# http://johnstantongeddes.org/assets/files/ApTranscriptome/sailfish_quant_20140916.tar
# check md5sum
md5sum sailfish_quant_20140916.tar
# 26102c7ef86cf30b5f8e923640378185

# move and extract
mkdir -p /results/trinity-full/sailfish-expression-Trinity-cap3-uclust
mv sailfish_quant_20140916.tar results/trinity-full/.
tar -xvf sailfish_quant_20140916.tar
```

For each sample, there is a directory containting a file *quant_bias_corrected.sf*. This file has the following columns, following a number of header lines:

1. Transcript ID
2. Transcript Length
3. Transcripts per Million (TPM): computed as described in (Li, Ruotti, Stewart, Thomson, and Dewey, 2009), and is meant as an estimate of the number of transcripts, per million observed transcripts, originating from each isoform.
4. Reads Per Kilobase per Million mapped reads (RPKM): classic measure of relative transcript abundance, and is an estimate of the number of reads per kilobase of transcript (per million mapped reads) originating from each transcript.

The TPM column for each sample was extracted and combined into a matrix for each species.

Preliminary examination of the data indicated that the A22_7 and Ar_7 samples may have been switched, so I remove these values from the combined expression data set for the two species. The 'Ar_7' sample clusters with all the other A22 samples, while the 'A22_7' sample clusters with all the other Ar samples. The parsimonious explanation for this is a labeling mistake. Removing these samples, rather than relabeling, is the conservative approach.

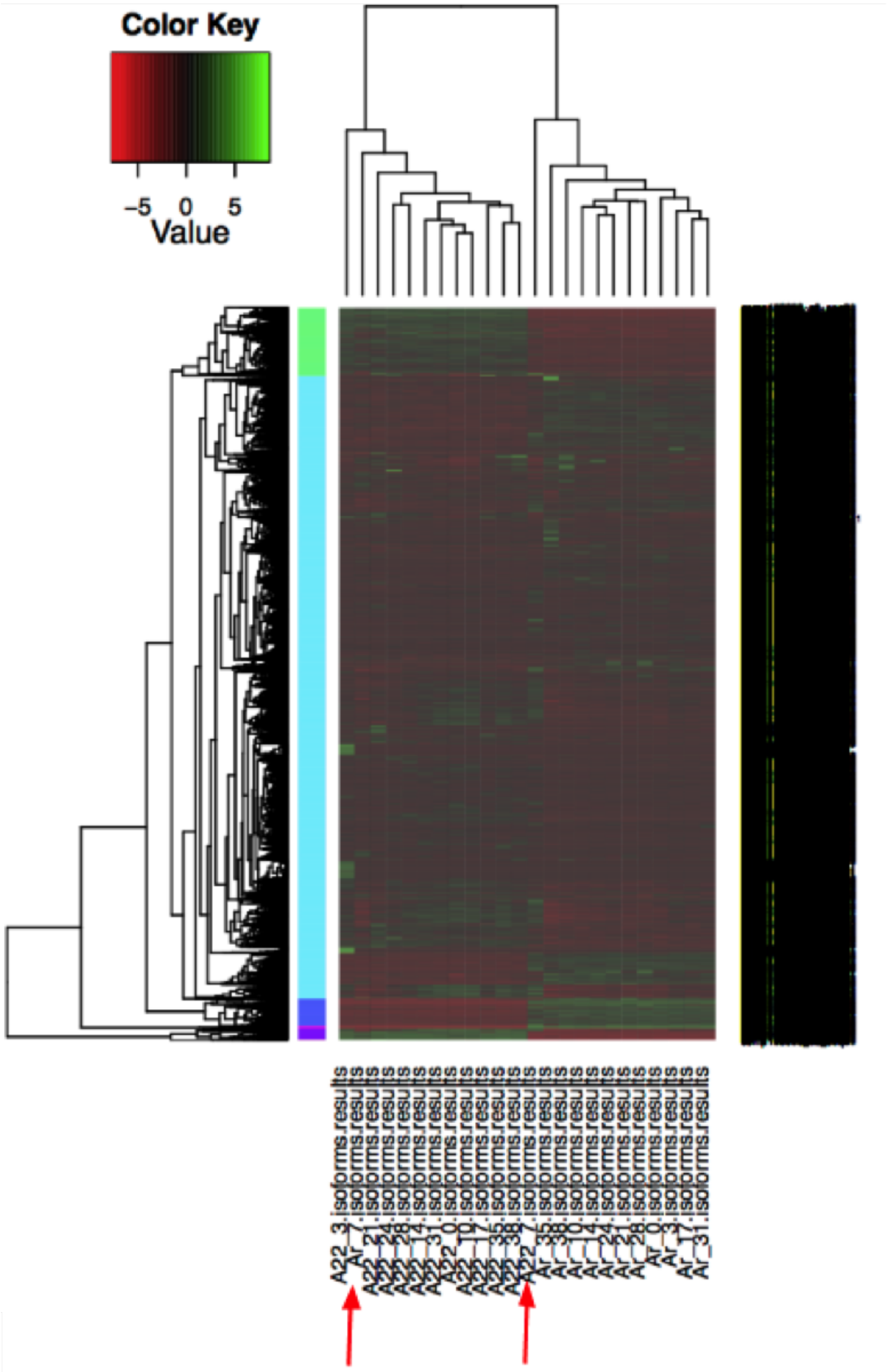
Color Key
−5 0 5
Value
A22_3.isoforms.results
Ar_31.isoforms.results

Note that expression levels at each temperature treatment are highly correlated between the two colonies, indicating common patterns of expression response to temperature.

Temperature	r
0	0.99
3.5	0.98
10.5	1
14	0.99
17.5	0.98
21	0.98
24.5	0.99
28	0.99
31.5	0.99
35	0.99
38.5	0.99

Table 3: Correlations between species for gene expression at temperature treatment

Remove *contaminant* transcripts

With gene expression quantified using all reads, I removed the transcripts identified as contaminants by DeconSeq.

```
# extract transcript names from fasta file rather than loading whole file
system("grep '^>' results/trinity-full/Trinity_cap3_uclust_cont.fa | cut -f 1 -d ' ' | sed 's/^.{1}//' >

# read file
cont.list <- read.table("results/trinity-full/cont.list")
# remote ">" leader
cont.list$V1 <- gsub(">", "", cont.list$V1)
str(cont.list)
```

```
## 'data.frame':    5675 obs. of  1 variable:
##  $ V1: chr  "225|*|Contig6496" "441|*|comp151639_c0_seq1" "2950|*|comp140219_c0_seq8" "3486|*|comp15
```

```
# remove from TPM.dt.sub
setkey(TPM.dt.sub, Transcript)
TPM.dt.sub <- TPM.dt.sub[!cont.list]
str(TPM.dt.sub)
```

```
## Classes 'data.table' and 'data.frame':   2160092 obs. of  10 variables:
##  $ Transcript       : chr  "0|*|Contig6267" "0|*|Contig6267" "0|*|Contig6267" "0|*|Contig6267" ...
##  $ Length           : int  9990 9990 9990 9990 9990 9990 9990 9990 9990 9990 ...
##  $ TPM              : num  0.079 0.0643 0.0357 0.093 0.0395 ...
##  $ RPKM             : num  0.0926 0.1078 0.0532 0.1418 0.0494 ...
```

```
##  $ KPKM             : num  0.0926 0.1078 0.0532 0.1418 0.0494 ...
##  $ EstimatedNumKmers: num  2974 1500 1373 2433 1713 ...
##  $ EstimatedNumReads: num  37.1 18.6 17.1 30.1 21.4 ...
##  $ sample           : chr  "A22-0" "Ar-0" "A22-3" "Ar-3" ...
##  $ val              : num  0 0 3.5 3.5 10.5 10.5 14 14 17.5 17.5 ...
##  $ colony           : Factor w/ 2 levels "A22","Ar": 1 2 1 2 1 2 1 2 1 2 ...
##  - attr(*, "sorted")= chr "Transcript"
##  - attr(*, ".internal.selfref")=<externalptr>
```

```
length(unique(TPM.dt.sub$Transcript))
```

```
## [1] 98186
```

```
# remove from annotation.table
setkey(annotation.table, Sequence.Name)
annotation.table <- annotation.table[!cont.list]
str(annotation.table)
```

```
## Classes 'data.table' and 'data.frame':   99861 obs. of  12 variables:
##  $ Sequence.Name        : chr  "0|*|Contig6267" "100000|*|comp2663136_c0_seq1" "100001|*|comp3439067_
##  $ sequence.length      : int  9990 208 208 208 208 208 208 208 208 208 ...
##  $ best.hit.to.nr       : chr  "gi|110756860|ref|XP_392375.3| PREDICTED: hypothetical protein LOC408
##  $ hit.length           : chr  "598" "-" "-" "69" ...
##  $ E.value              : chr  "2.1e-265" "-" "-" "5.11e-40" ...
##  $ Bit.score            : chr  "904.618736" "-" "-" "161.159029" ...
##  $ GO.Biological.Process: chr  "GO:0035335 peptidyl-tyrosine dephosphorylation | GO:0000188 inactiva
##  $ GO.Cellular.Component: chr  "-" "-" "-" "GO:0005840 ribosome" ...
##  $ GO.Molecular.Function: chr  "GO:0017017 MAP kinase tyrosine/serine/threonine phosphatase activity
##  $ Enzyme               : chr  "3.1.3.16  | 3.1.3.48 " "-" "-" "-" ...
##  $ Domain               : chr  "pfam00782 DSPc | pfam00581 Rhodanese" "pfam03993 DUF349" "-" "pfam00
##  $ annotation.type      : chr  "GO & Enzyme & Domain" "Domain only" "" "GO & Domain" ...
##  - attr(*, "sorted")= chr "Sequence.Name"
##  - attr(*, ".internal.selfref")=<externalptr>
```

Regression-model to identify thermally-responsive genes

To identify transcripts (roughly equivalent to genes) that show thermal responsiveness, I fit the following linear model to each transcript:

$$log(TPM + 1) = \beta_0 + \beta_1(species) + \beta_2(temp) + \beta_3(temp^2) + \beta_4(species * temp) + \beta_5(species * temp^2) + \epsilon$$

where TPM is transcripts per million.

(1) Identify transcripts with overall significant model fit. Adjust P values for multiple testing using FDR and retain transcripts with FDR < 0.05. Use log-transformed response to account for outliers.

```
# define model for RxN function
model <-  "log(TPM+1) ~ colony + val + I(val^2) + colony:val + colony:I(val^2)"

# calculate overall P value and R^2 for each transcript
RxNpval <- ddply(TPM.dt.sub, .(Transcript), .inform="TRUE", modpFunc)
```

Of the 98186 transcripts, 22089 have models with P < 0.05.

Many of these are likely false positives, so I adjusted P-values using false discovery rate (FDR).

```
RxNpval$padj <- p.adjust(RxNpval$pval, method = "fdr")
# Plot FDR values against initial pvalues
par(mfrow = c(2,1))
hist(RxNpval$pval)
hist(RxNpval$padj)
```

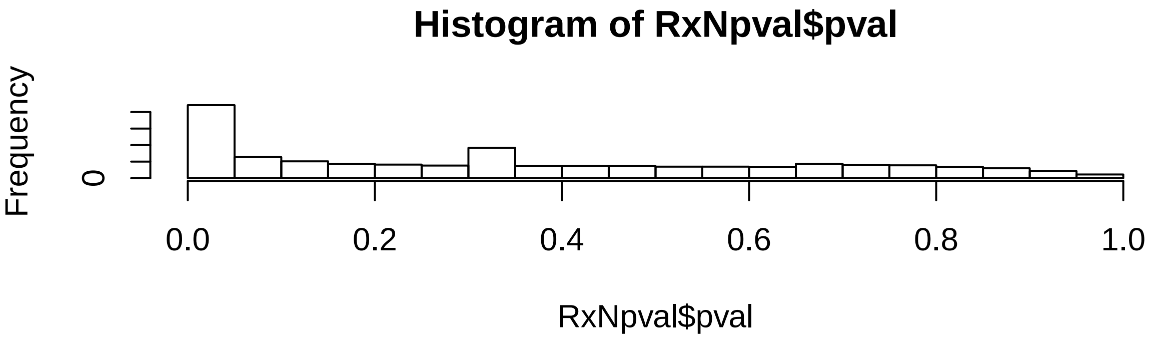

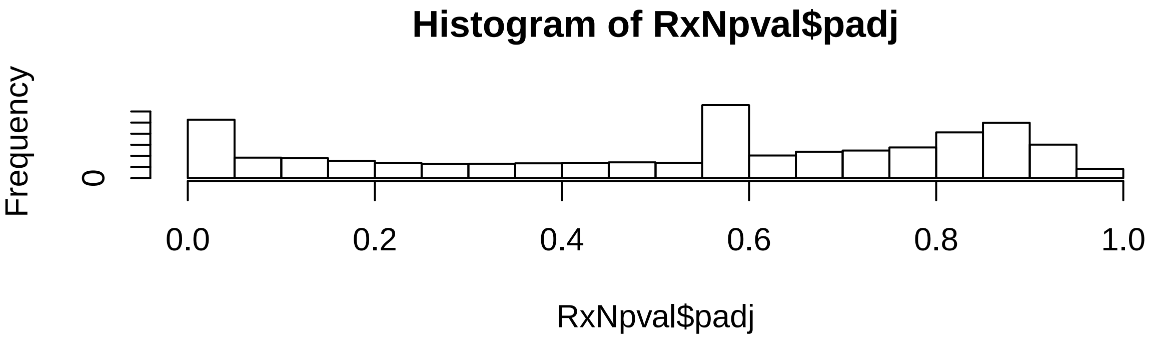


```
# subset to significant transcripts
pre.signif.transcripts <- RxNpval[which(RxNpval$padj < 0.05), ]
```

At the 5% FDR significance threshold, there are 10525 transcripts with an overall significant model.

Feedback from reviewers questioned whether this was sufficient to remove all false positives. To examine this, we performed a resampling simulation to determine how many transcripts would be identified as significant using the above approach. Specifically, we randomly re-assigned the expression levels to the temperature treatments for each species, fit the same linear model as for the real data, calculated the number of significant transcripts under the 5% FDR threshold, and repeated this 100 times. We then took the 95th percentile of the distribution of significant transcripts from the resampled datasets as the true null level of false positives.

As this resampling is slow, I ran it in a separate file (ApTranscriptome_resample.Rmd) and load the results here.

```
# load resampling results
load("data/resample.Rda")
```

Using this approach, the empirical false positive, *after* adjusting P-values for FDR, is 8016, indicating that only 2509 are true positives.

```r
# order by padj
pre.signif.transcripts <- pre.signif.transcripts[order(pre.signif.transcripts$padj), ]
# number to keep
keep <- nrow(pre.signif.transcripts) - quantile(D$num_signif, probs = 0.95)
signif.transcripts <- pre.signif.transcripts[1:keep, ]

# extract significant transcripts
sig.TPM.dt.sub <- TPM.dt.sub[signif.transcripts$Transcript]
```

(2) Fit linear model to significantly responsive transcripts, perform stepAIC to retain only significant terms, and save `lm` output to list

```r
# perform model selection for responsive transcripts
# need to use `try` to avoid stopping on error for AIC at Infinity
RxNlmAIC <- try(dlply(sig.TPM.dt.sub, .(Transcript), lmFunc))
```

The set of transcripts with significant expression patterns include those with expression that differs by species, temperature and the interaction of species and temperature. I am specifically interested in the thermally-responsive transcripts (temperature and species x temperature) so I subset the significant transcripts to examine these.

```r
interaction.lms <- RxNlmAIC[which(Map(grepFunc, RxNlmAIC, term = "colonyAr:") == TRUE)]
other.lms <- RxNlmAIC[setdiff(names(RxNlmAIC), names(interaction.lms))]
temperature.lms <- other.lms[which(Map(grepFunc, other.lms, term = "val") == TRUE)]
colony.lms <- other.lms[setdiff(names(other.lms), names(temperature.lms))]

# combine all responsive transcripts
responsive.lms <- c(temperature.lms, interaction.lms)
rm(other.lms)

quadratic.lms <- RxNlmAIC[which(Map(grepFunc, RxNlmAIC, term = "2") == TRUE)]
```

Coefficient	Number.significant
Total	2,509
Colony	431
Temperature	525
Temperature:Colony	1,553

Table 4: Number of transcripts with expression that depends on species, temperature or their interaction at 5% FDR out of 98,186 total transcripts.

Thermal-response expression categories

The previous section identified the transcripts with thermally-responsive expression. In this section, I determined the shape of the expression response to temperature for each transcript. Categories of expression response are:

- **High** - increase expression with temperature
- **Low** - decrease expression with temperature
- **Intermediate** - maximum expression at intermediate temperatures (14 - 28C)
- **Bimodal** - expressed greater than one standard deviation of expression at both low and high temperatures
- **Not Responsive** - not thermally-responsive in one species but does respond in the other

For the transcripts where thermal-responsive expression depends on species, I determined the functional type of the expression response separately for each species.

```
# calculate response type for all temperature responsive transcripts
sig.response.type <- ldply(responsive.lms, .progress = "none", .inform = TRUE, RxNtype)
colnames(sig.response.type)[which(colnames(sig.response.type) == ".id")] <- "Transcript"
sig.response.type <- data.table(sig.response.type)
setkey(sig.response.type, Transcript)
str(sig.response.type)
```

```
## Classes 'data.table' and 'data.frame':   2078 obs. of  9 variables:
##  $ Transcript: chr  "100148|*|comp125464_c0_seq1" "100636|*|comp3558092_c0_seq1" "100709|*|comp14774
##  $ A22.max   : num  0 0 38.5 0 0 0 0 38.5 17.5 0 ...
##  $ A22.min   : num  23 26 0 26 38.5 38.5 38.5 0.5 38.5 26 ...
##  $ A22.opt   : num  5.096 0.953 1.093 0.947 1149.236 ...
##  $ A22.type  : chr  "Bimodal" "Low" "High" "Low" ...
##  $ Ar.max    : num  18 NA 0 NA 0 38.5 0 0 38.5 NA ...
##  $ Ar.min    : num  38.5 NA 38.5 NA 38.5 0 38.5 38.5 12.5 NA ...
##  $ Ar.opt    : num  1.01 1 4.56 1 1.24 ...
##  $ Ar.type   : chr  "Intermediate" "NotResp" "Low" "NotResp" ...
##  - attr(*, ".internal.selfref")=<externalptr>
##  - attr(*, "sorted")= chr "Transcript"
```

```
# calculate response type for transcripts with species x temperature interaction only
interaction.response.type <- ldply(interaction.lms, .progress = "none", .inform = TRUE, RxNtype)
colnames(interaction.response.type)[which(colnames(interaction.response.type) == ".id")] <- "Transcript
interaction.response.type <- data.table(interaction.response.type)
setkey(interaction.response.type, Transcript)
str(interaction.response.type)
```

```
## Classes 'data.table' and 'data.frame':   1553 obs. of  9 variables:
##  $ Transcript: chr  "100148|*|comp125464_c0_seq1" "100636|*|comp3558092_c0_seq1" "100709|*|comp14774
##  $ A22.max   : num  0 0 38.5 0 0 38.5 17.5 0 0 0 ...
##  $ A22.min   : num  23 26 0 26 38.5 0.5 38.5 26 38.5 25 ...
##  $ A22.opt   : num  5.096 0.953 1.093 0.947 3.192 ...
##  $ A22.type  : chr  "Bimodal" "Low" "High" "Low" ...
##  $ Ar.max    : num  18 NA 0 NA 38.5 0 38.5 NA 18 21 ...
##  $ Ar.min    : num  38.5 NA 38.5 NA 0 38.5 12.5 NA 38.5 0 ...
##  $ Ar.opt    : num  1.01 1 4.56 1 1.04 ...
##  $ Ar.type   : chr  "Intermediate" "NotResp" "Low" "NotResp" ...
##  - attr(*, ".internal.selfref")=<externalptr>
##  - attr(*, "sorted")= chr "Transcript"
```

```
# calculate response types for transcripts without interactions
temperature.response.type <- ldply(temperature.lms, .progress = "none", .inform = TRUE, RxNtype)

# save results to file
write.table(file = paste(resultsdir, "Ap_responsive_transcripts_", Sys.Date(), ".txt", sep = ""), sig.re
```

Next, I compared the number of thermally-responsive in each response category between the two colonies.

```
A22.type.table <- table(sig.response.type$A22.type)
Ar.type.table <- table(sig.response.type$Ar.type)

# table
(Ap.type.table <- rbind(A22.type.table, Ar.type.table))
```

```
##                Bimodal High Intermediate  Low NotResp
## A22.type.table     278  248          249 1193     110
## Ar.type.table      117  232          680  920     129
```

```
# proportion
Ap.type.table.prop <- round(Ap.type.table / (sum(Ap.type.table)/2) * 100)

# test if the number of transcripts in each category differs from null expectations using Pearson Chi-s
(chi1 <- chisq.test(Ap.type.table))
```

```
##
##  Pearson's Chi-squared test
##
## data:  Ap.type.table
## X-squared = 300, df = 4, p-value <2e-16
```

```
# test if the total number of responsive transcripts differs between species
resp.table <- matrix(nrow=2, ncol=2, dimnames = list(c("R", "NR"), c("Ap", "Ac")))
resp.table[1,1] <- sum(A22.type.table[which(names(A22.type.table) != "NotResp")])
resp.table[2,1] <- nrow(annotation.table) - sum(A22.type.table[which(names(A22.type.table) != "NotResp"
resp.table[1,2] <- sum(Ar.type.table[which(names(Ar.type.table) != "NotResp")])
resp.table[2,2] <- nrow(annotation.table) - sum(Ar.type.table[which(names(Ar.type.table) != "NotResp")]
resp.table
```

```
##       Ap    Ac
## R   1968  1949
## NR 97893 97912
```

```
(chi2 <- chisq.test(resp.table))
```

```
##
##  Pearson's Chi-squared test with Yates' continuity correction
##
## data:  resp.table
## X-squared = 0.08, df = 1, p-value = 0.8
```

```r
# test if the number of Low responsive transcripts differs between species
low.table <- matrix(nrow=2, ncol=2, dimnames = list(c("Low", "NotLow"), c("Ap", "Ac")))
low.table[1,1] <- A22.type.table[which(names(A22.type.table) == "Low")]
low.table[2,1] <- sum(A22.type.table[which(names(A22.type.table) != "Low")])
low.table[1,2] <- Ar.type.table[which(names(Ar.type.table) == "Low")]
low.table[2,2] <- sum(Ar.type.table[which(names(Ar.type.table) != "Low")])
low.table
```

```
##          Ap   Ac
## Low    1193  920
## NotLow  885 1158
```

```r
(chi3 <- chisq.test(low.table))
```

```
##
##  Pearson's Chi-squared test with Yates' continuity correction
##
## data:  low.table
## X-squared = 70, df = 1, p-value <2e-16
```

```r
# test if the number of Intermediate responsive transcripts differs between species
intermediate.table <- matrix(nrow=2, ncol=2, dimnames = list(c("Intermediate", "Others"), c("Ap", "Ac")
intermediate.table[1,1] <- A22.type.table[which(names(A22.type.table) == "Intermediate")]
intermediate.table[2,1] <- sum(A22.type.table[which(names(A22.type.table) != "Intermediate")])
intermediate.table[1,2] <- Ar.type.table[which(names(Ar.type.table) == "Intermediate")]
intermediate.table[2,2] <- sum(Ar.type.table[which(names(Ar.type.table) != "Intermediate")])
intermediate.table
```

```
##                Ap   Ac
## Intermediate  249  680
## Others       1829 1398
```

```r
(chi4 <- chisq.test(intermediate.table))
```

```
##
##  Pearson's Chi-squared test with Yates' continuity correction
##
## data:  intermediate.table
## X-squared = 300, df = 1, p-value <2e-16
```

```r
# test if the number of High responsive transcripts differs between species
high.table <- matrix(nrow=2, ncol=2, dimnames = list(c("High", "Others"), c("Ap", "Ac")))
high.table[1,1] <- A22.type.table[which(names(A22.type.table) == "High")]
high.table[2,1] <- sum(A22.type.table[which(names(A22.type.table) != "High")])
high.table[1,2] <- Ar.type.table[which(names(Ar.type.table) == "High")]
high.table[2,2] <- sum(Ar.type.table[which(names(Ar.type.table) != "High")])
high.table
```

```
##          Ap   Ac
## High    248  232
## Others 1830 1846
```

```r
(chi5 <- chisq.test(high.table))
```

```
##
##  Pearson's Chi-squared test with Yates' continuity correction
##
## data:  high.table
## X-squared = 0.5, df = 1, p-value = 0.5
```

```r
# test if the number of Bimodal responsive transcripts differs between species
bimodal.table <- matrix(nrow=2, ncol=2, dimnames = list(c("Bimodal", "Others"), c("Ap", "Ac")))
bimodal.table[1,1] <- A22.type.table[which(names(A22.type.table) == "Bimodal")]
bimodal.table[2,1] <- sum(A22.type.table[which(names(A22.type.table) != "Bimodal")])
bimodal.table[1,2] <- Ar.type.table[which(names(Ar.type.table) == "Bimodal")]
bimodal.table[2,2] <- sum(Ar.type.table[which(names(Ar.type.table) != "Bimodal")])
bimodal.table
```

```
##           Ap   Ac
## Bimodal  278  117
## Others  1800 1961
```

```r
(chi6 <- chisq.test(bimodal.table))
```

```
##
##  Pearson's Chi-squared test with Yates' continuity correction
##
## data:  bimodal.table
## X-squared = 70, df = 1, p-value <2e-16
```

The total number of responsive transcripts does not differ between species, though their groupings into expression categories does differ.

Test of marginal frequencies between transcripts in each expression category between species

The question of biological interest is whether the marginal frequencies differ between the two species. Statistically, this can be addressed using the generalized [McNemar's test](http://en.wikipedia.org/wiki/McNemar's_test) of marginal homogeneity.

```r
type.table <- table(Acar = sig.response.type$Ar.type, Apic = sig.response.type$A22.type)
# reorder
tt2 <- type.table[c("Low", "Intermediate", "High", "Bimodal", "NotResp"), c("Low", "Intermediate", "Hig

# McNemar's test
mh.test <- mh_test(as.table(tt2))

# calculate contribution of each off-diagonal to Z~0~
d1 <- sum(tt2[1,2:5]) - sum(tt2[2:5,1])
d2 <- sum(tt2[2,c(1,3:5)]) - sum(tt2[c(1,3:5),2])
d3 <- sum(tt2[3,c(1:2,4:5)]) - sum(tt2[c(1:2,4:5),3])
d4 <- sum(tt2[4,c(1:3,5)]) - sum(tt2[c(1:3,5),4])
```

```r
d5 <- sum(tt2[5,1:4]) - sum(tt2[1:4,5])

# proportion of test statistic due to off-diagonals
dsum <- sum(abs(d1), abs(d2), abs(d3), abs(d4), abs(d5))
abs(d1)/dsum
```

```
## [1] 0.303
```

```r
abs(d2)/dsum
```

```
## [1] 0.479
```

```r
abs(d3)/dsum
```

```
## [1] 0.0178
```

```r
abs(d4)/dsum
```

```
## [1] 0.179
```

```r
abs(d5)/dsum
```

```
## [1] 0.0211
```

```r
# e2 (Acar Intermediate) contributes ~48% to Z~0~
```

To identify specific cells that deviate from null expectation, I calculated the expected observations assuming that marginal frequencies equal the overall frequency for each expression category, then determined which cells have deviations between observed and expected that are greater than the overall X^2 statistic.

```r
# overall mean for each class
rs <- rowSums(tt2)
cs <- colSums(tt2)
(gm <- (rs + cs) / sum(tt2 * 2))
```

```
##          Low Intermediate         High      Bimodal      NotResp
##       0.5084       0.2235       0.1155       0.0950       0.0575
```

```r
# calculate observed values for each cell using overall mean for each expression type
Ec <- outer(gm, gm, "*") * sum(tt2)

# get deviations of observed from expected
Ec.dev <- tt2 - Ec

# calculate chi-squared deviation
Ec.cells <- sign(tt2 - Ec) * (tt2 - Ec)^2 / Ec

# which of these are significantly great than expected chi-squared?
```

To visualize this, I made a heatmap that showed the deviation of the actual number of transcripts in each cell from the expected count.

```
md <- reshape2::melt(Ec.dev)

mh_plot <- qplot(x=Apic, y=Acar, data=md, fill=value, geom="tile", ylim = rev(levels(md$Acar))) +
  scale_fill_gradient2(limits=c(-200, 200), low="#f1a340", high="#998ec3") +
  theme(axis.title = element_text(face="italic")) +
  labs(x = "A. picea", y = "A. carolinensis")

mh_plot
```

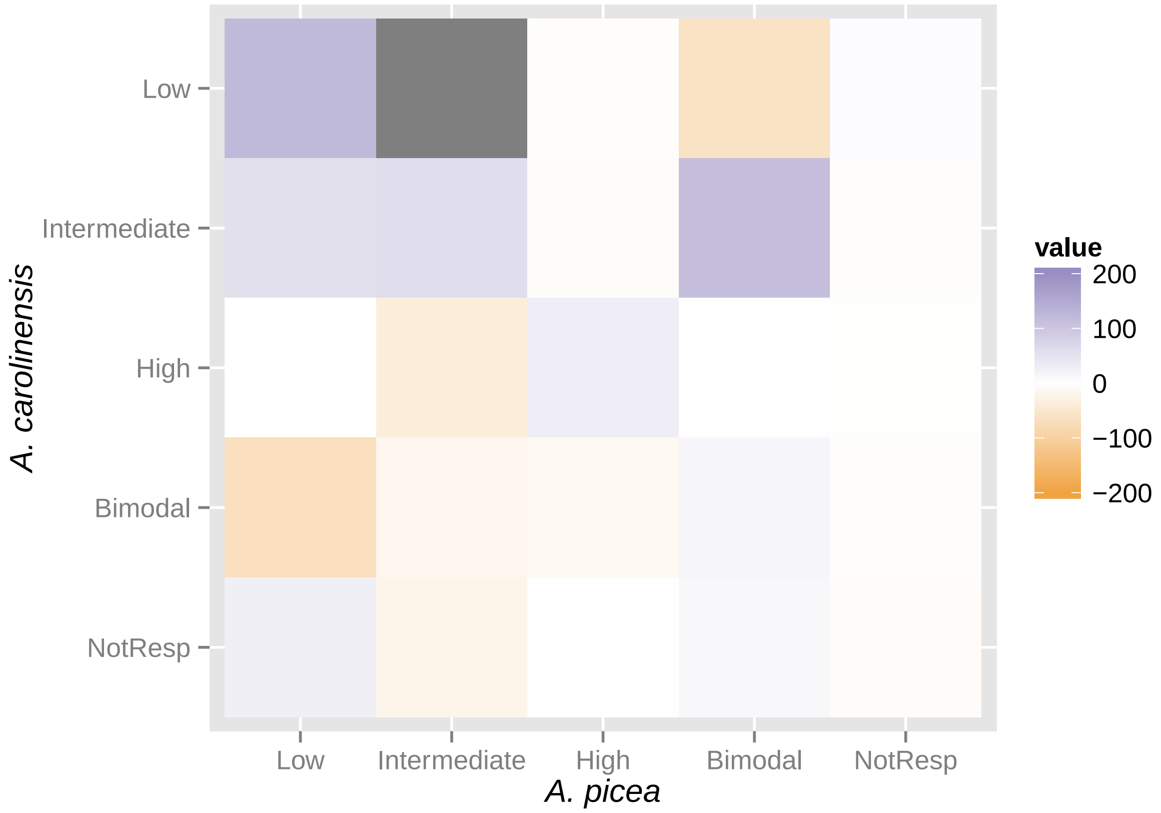


```
# make subplot of rows that match hypotheses

# A. carolinensis **Low** and **Bimodal**
md_sub1 <- subset(md, Acar %in% c("Bimodal", "Low"), )
md_sub1 <- droplevels(md_sub1)

mh_plot_sub1 <- qplot(y=Apic, x=Acar, data=md_sub1, fill=value, geom="tile", xlim = rev(levels(md_sub1$A
  scale_fill_gradient2(limits=c(-200, 200), low="#f1a340", high="#998ec3") +
  theme(axis.title = element_text(face="italic")) +
  labs(y = "A. picea", x = "A. carolinensis") +
  theme(legend.position = "right") +
  geom_rect(xmin = 0.5, xmax = 1.5, ymin = 3.5, ymax = 4.5, fill = "transparent", linetype=2, colour="bl
  geom_rect(xmin = 1.5, xmax = 2.5, ymin = 0.5, ymax = 1.5, fill = "transparent", linetype=2, colour="bl

mh_plot_sub1
```

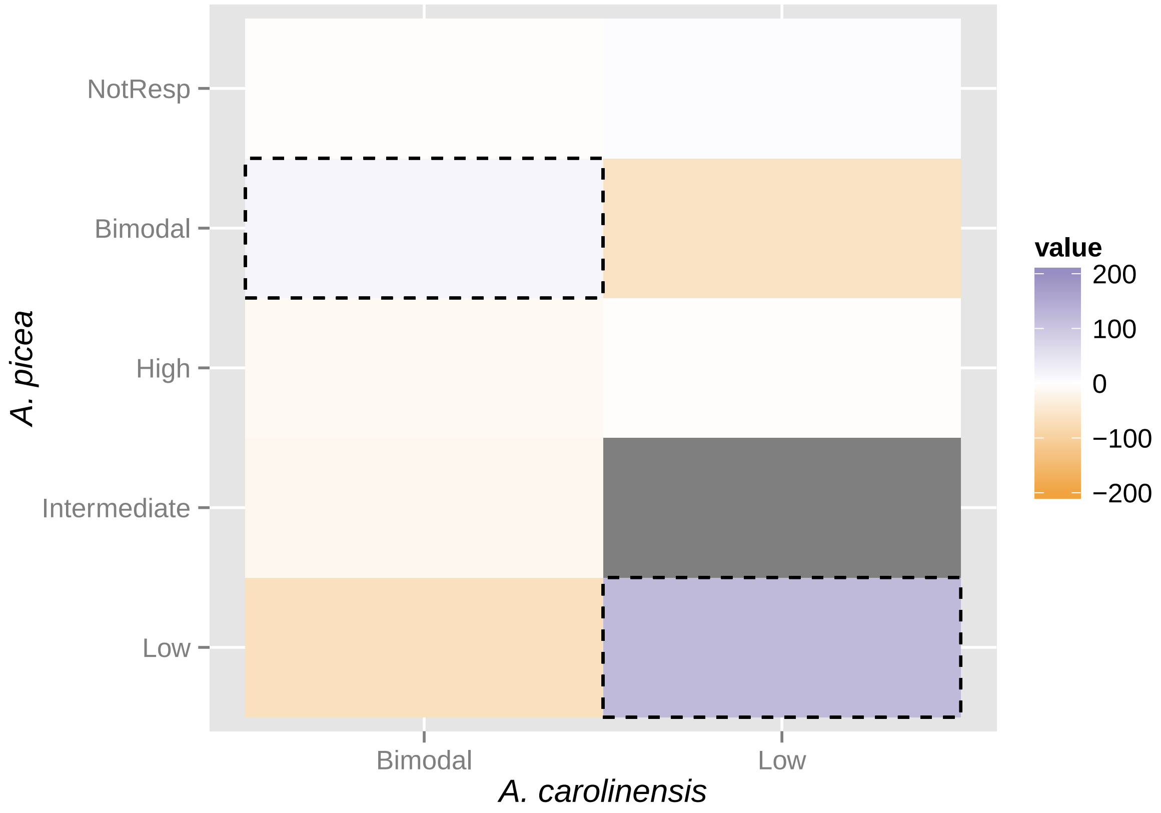


```
# A. picea **High** and **Bimodal**
md_sub2 <- subset(md, Apic %in% c("High", "Bimodal"), )
md_sub2 <- droplevels(md_sub2)

mh_plot_sub2 <- qplot(y=Acar, x=Apic, data=md_sub2, fill=value, geom="tile", xlim = rev(levels(md_sub2$A
  scale_fill_gradient2(limits=c(-200, 200), low="#f1a340", high="#998ec3") +
  theme(axis.title = element_text(face="italic")) +
  labs(y = "A. carolinensis", x = "A. picea") +
  theme(legend.position = "none") +
  geom_rect(xmin = 0.5, xmax = 1.5, ymin = 3.5, ymax = 4.5, fill = "transparent", linetype=2, colour="bl
  geom_rect(xmin = 1.5, xmax = 2.5, ymin = 2.5, ymax = 3.5, fill = "transparent", linetype=2, colour="bl

mh_plot_sub2
```

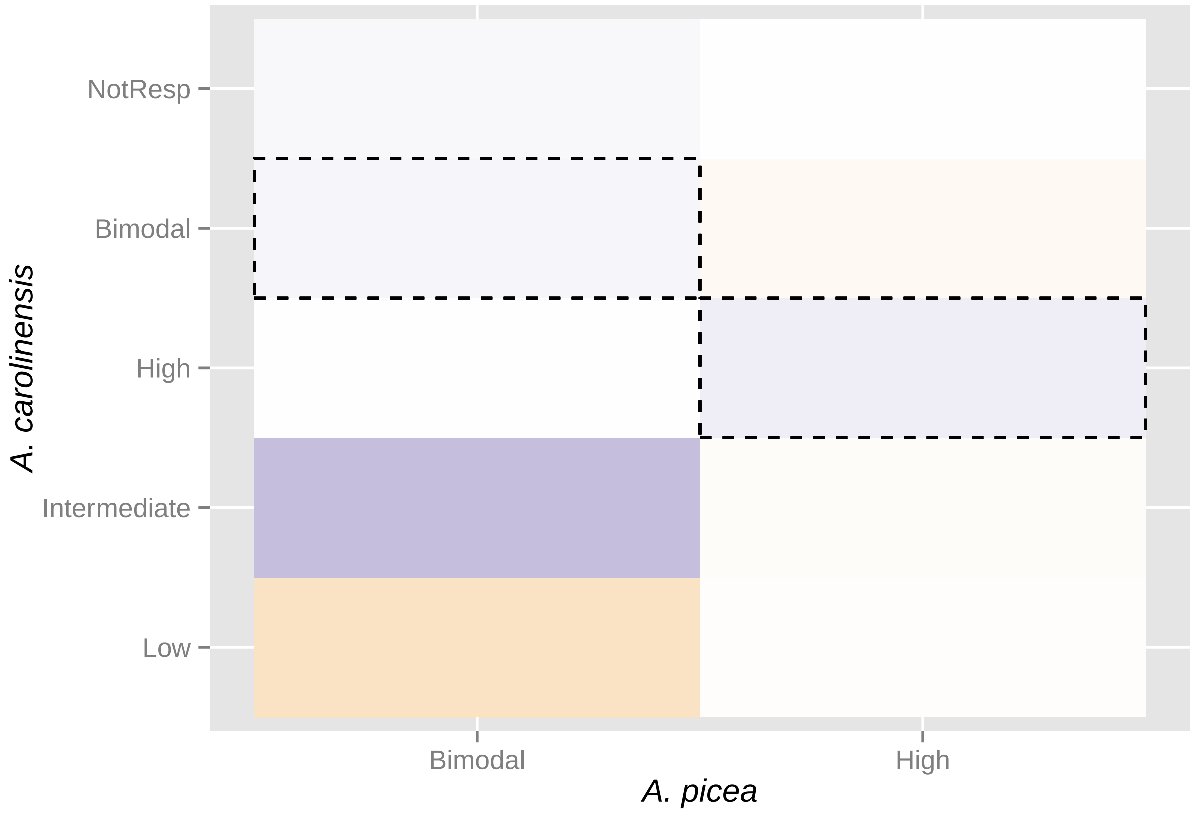


```
# arrange plots to make figure for manuscript
mh_plot_sub1 <- mh_plot_sub1 + ggtitle('A') + theme(plot.title = element_text(hjust=0))
mh_plot_sub2 <- mh_plot_sub2 + ggtitle('B') + theme(plot.title = element_text(hjust=0))

png("results/matched_observations_Fig2.png")
grid.arrange(mh_plot_sub1, mh_plot_sub2, ncol=2, widths = c(3.75, 3))
dev.off()
```

```
## pdf
##   2
```

```
# full figure for supplemental
png("results/matched_observations_FigS1.png")
mh_plot
dev.off()
```

```
## pdf
##   2
```

The *enhanced response hypothesis* posits that temperature adaptation uses existing response mechanisms, which should result in significant overlap in response. This pattern would be apparent as an excess of transcripts with the same response pattern between species (e.g. more shared “Low” transcripts) and a deficit of transcripts that shift to other response patterns or become not responsive.

```
enhanced_response_dat <- md_sub1
enhanced_response_dat$value <- 0
```

```r
# set non-shared response to random negative value
enhanced_response_dat[which(enhanced_response_dat$Acar == "Low"), "value"] <- runif(5, min=-20, max=-10
enhanced_response_dat[which(enhanced_response_dat$Acar == "Bimodal"), "value"] <- runif(5, min=-20, max
# change shared response to positive
enhanced_response_dat[which(enhanced_response_dat$Acar == "Low" & enhanced_response_dat$Apic == "Low"),
enhanced_response_dat[which(enhanced_response_dat$Acar == "Bimodal" & enhanced_response_dat$Apic == "Bi

enhanced_response_dat
```

```
##       Acar         Apic value
## 1      Low          Low  30.0
## 4  Bimodal          Low -17.1
## 6      Low Intermediate -18.3
## 9  Bimodal Intermediate -14.5
## 11     Low         High -13.7
## 14 Bimodal         High -11.5
## 16     Low      Bimodal -13.8
## 19 Bimodal      Bimodal  30.0
## 21     Low      NotResp -19.1
## 24 Bimodal      NotResp -16.3
```

```r
# plot
enhanced_response_plot <- ggplot(enhanced_response_dat, aes(y = Apic, x = Acar, fill=value)) +
  geom_tile(colour = "#ffffff", size =1) +
  xlim(rev(levels(enhanced_response_dat$Acar))) +
  scale_fill_gradient2(limits=c(-100, 100), low="#f1a340", high="#998ec3") +
  theme(axis.title = element_text(face="italic")) +
  labs(y = "A. picea", x = "A. carolinensis") +
  theme(legend.position = "right") +
  geom_rect(xmin = 0.5, xmax = 1.5, ymin = 3.5, ymax = 4.5, fill = "transparent", linetype=2, colour="bl
  geom_rect(xmin = 1.5, xmax = 2.5, ymin = 0.5, ymax = 1.5, fill = "transparent", linetype=2, colour="bl

# title
enhanced_response_plot <- enhanced_response_plot + ggtitle('Enhanced Response hypothesis') + theme(plot
enhanced_response_plot
```

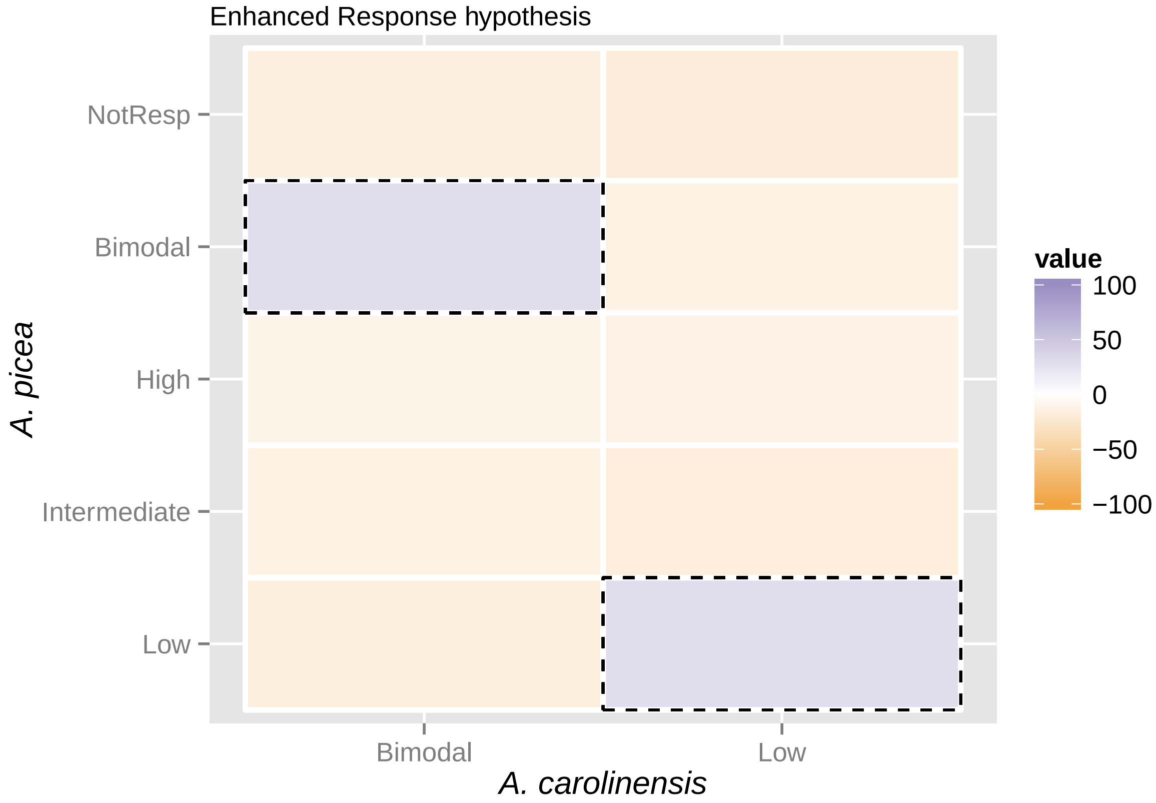

In contrast, the *tolerance hypothesis* predicts that genes involved in active response will become non-responsive or shift to other response categories in the better-adapted species.

```
tolerance_dat <- md_sub1
tolerance_dat$value <- 0
# set non-shared response to random positive value
tolerance_dat[which(tolerance_dat$Acar == "Low"), "value"] <- runif(5, min=40, max=60)
tolerance_dat[which(tolerance_dat$Acar == "Bimodal"), "value"] <- runif(5, min=40, max=60)
# change shared response to near zero
tolerance_dat[which(tolerance_dat$Acar == "Low" & tolerance_dat$Apic == "Low"), "value"] <- -20
tolerance_dat[which(tolerance_dat$Acar == "Bimodal" & tolerance_dat$Apic == "Bimodal"), "value"] <- -20

tolerance_dat
```

```
##       Acar         Apic value
## 1      Low          Low -20.0
## 4  Bimodal          Low  45.2
## 6      Low Intermediate  47.2
## 9  Bimodal Intermediate  53.4
## 11     Low         High  58.6
## 14 Bimodal         High  46.5
## 16     Low      Bimodal  56.6
## 19 Bimodal      Bimodal -20.0
## 21     Low      NotResp  40.2
## 24 Bimodal      NotResp  51.1
```

```r
# plot
tolerance_plot <- ggplot(tolerance_dat, aes(y=Apic, x=Acar, fill=value)) +
  geom_tile(colour = "#ffffff", size =1) +
  xlim(rev(levels(enhanced_response_dat$Acar))) +
  scale_fill_gradient2(limits=c(-100, 100), low="#f1a340", high="#998ec3") +
  theme(axis.title = element_text(face="italic")) +
  labs(y = "A. picea", x = "A. carolinensis") +
  theme(legend.position = "none") +
  geom_rect(xmin = 0.5, xmax = 1.5, ymin = 3.5, ymax = 4.5, fill = "transparent", linetype=2, colour="bl
  geom_rect(xmin = 1.5, xmax = 2.5, ymin = 0.5, ymax = 1.5, fill = "transparent", linetype=2, colour="bl

# title
tolerance_plot <- tolerance_plot + ggtitle('Tolerance hypothesis') + theme(plot.title = element_text(hj
tolerance_plot
```

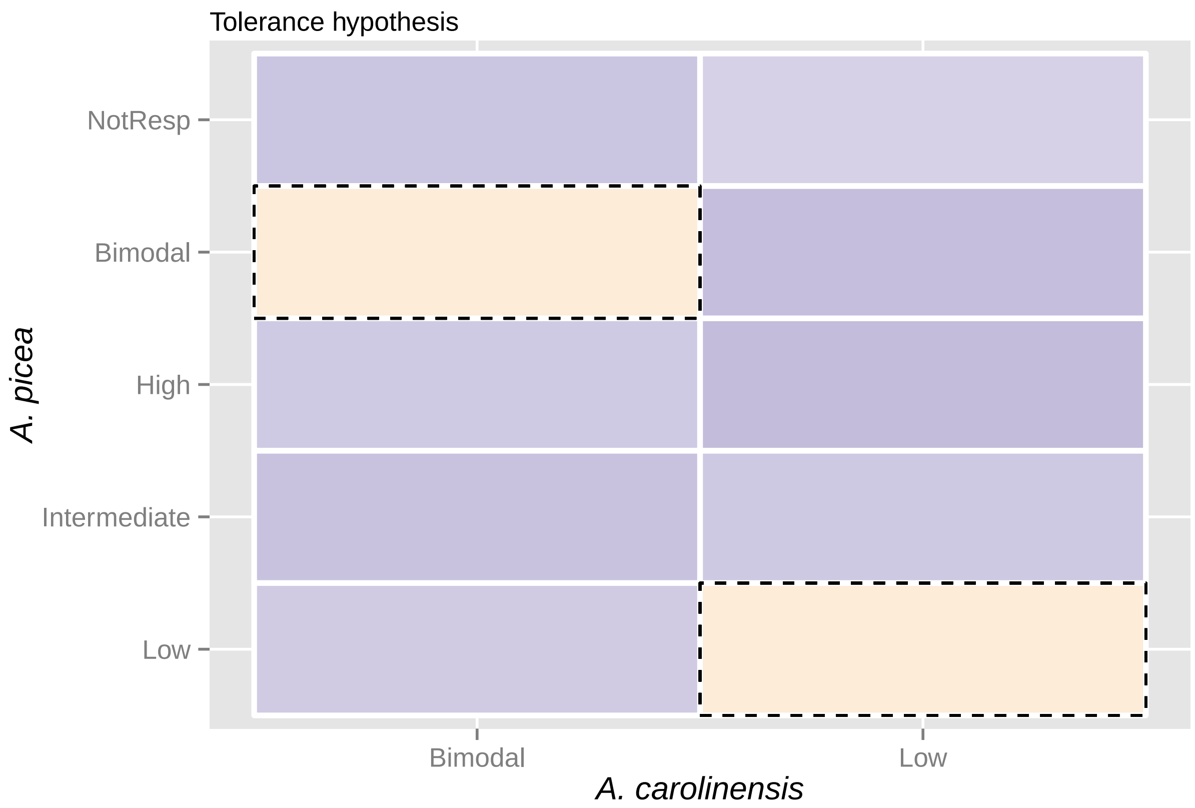


```r
# plot together
png("results/predictions_Fig1.png")
grid.arrange(enhanced_response_plot, tolerance_plot, ncol=2, widths = c(3.7, 3))
dev.off()
```

```
## pdf
##   2
```

The number of thermally-responsive in each response category differed between the colonies. For *A. picea*, the majority of the responsive transcripts were in the *Low* category, followed by *Bimodal*, *High*, *Intermediate* and *Not Responsive*. For *A. carolinensis*, the majority of responsive transcripts were in the *Bimodal* and *High* categories, followed by *Intermediate*, *Low* and *Not Responsive*.

Interestingly, over half of the *High* genes in *AcNC* are *Low* in *ApVT*, and vice versa. In contrast, most of the *Low* genes in one species are also *Low* in the other species.

	**ApVT**						
**AcNC**		Low	Intermediate	High	Bimodal	NotResp	Total
	Low	659	36	120	39	66	920
	Intermediate	290	163	48	156	23	680
	High	122	17	57	24	12	232
	Bimodal	34	28	10	36	9	117
	NotResp	88	5	13	23	0	129
	Total	1193	249	248	278	110	2078

Table 5: Number of transcripts with maximum expression at high, low, intermediate, both high and low (bimodal) temperatures or are not thermally-responsivefor each species and their overlap.

Table 4 shows the number of transcripts that fall into each expression type for each each species. The totals for each species *do not* include the 525 transcripts that have consistent temperature responses between the two colonies.

An interesting observation from the matched observations plot (Fig. 1) is that for **NotResp** genes in *A. carolinensis* the **Bimodal** category in *A. picea* is over-represented. Finding that the mean expression level of these genes in *A. carolinensis* is greater than the expression level at the rearing temperature (25°C) in *A. picea* would be consistent with genetic assimilation. That is, they have evolved to be constitutively activated in *A. carolinensis* and thus are no longer thermally-responsive as they are in *A. picea*.

```
Ap.bim.Ac.nr <- sig.response.type[which(sig.response.type$A22.type == "Bimodal" & sig.response.type$Ar.t

# get mean expression level for Ar transcripts
Ap.bim.Ac.nr.TPM <- TPM.dt.sub[Ap.bim.Ac.nr$Transcript]
Ap.bim.Ac.nr.mean.exp.NR <- ddply(Ap.bim.Ac.nr.TPM, .(Transcript), summarize, meanTPM = mean(TPM))

# get expression at optimum for A22 transcripts
Ap.bim.Ac.nr.opt.exp <- Ap.bim.Ac.nr$A22.opt

t.test(Ap.bim.Ac.nr.mean.exp.NR$meanTPM, Ap.bim.Ac.nr.opt.exp)
```

```
##
##  Welch Two Sample t-test
##
## data:  Ap.bim.Ac.nr.mean.exp.NR$meanTPM and Ap.bim.Ac.nr.opt.exp
## t = -10, df = 30, p-value = 2e-12
## alternative hypothesis: true difference in means is not equal to 0
## 95 percent confidence interval:
##  -1.222 -0.836
## sample estimates:
## mean of x mean of y
##     0.199     1.228
```

This hypothesis was not supported as expression was higher for the **Bimodal** transcripts in *A. picea* than the non-responsive *A. carolinensis* transcripts.

Species comparisons

In this section, I performed a number of comparisons of the thermal reactionomes between the *Ar* and *A22* species. Specifically, I:

- compared profiles of the temperature of maximum expression for thermally-responsive transcripts
- compared basal expression at optimal temperature for thermally-responsive genes
- compared degree of upregulation to constitutive expression
- compared the critical temperature of transcript upregulation, T_{on}, between species for transcripts induced in response to high or low temperatures
- compared the critical temperature of transcript downregulation, T_{off}, between species for transcripts induced in response to high or low temperatures

For these tests, I predicted expression for responsive transcripts.

```
# subset to transcripts with significant temperature x species interaction
resp.TPM.dt.sub <- TPM.dt.sub[names(interaction.lms)]

# apply predFunc to all responsive transcripts
resp.TPM.dt.sub.pred <- ddply(resp.TPM.dt.sub, .(Transcript), .inform="TRUE", predFunc)

# setkey to Transcript and colony
resp.TPM.dt.sub.pred <- data.table(resp.TPM.dt.sub.pred)
setkey(resp.TPM.dt.sub.pred, Transcript, colony)
```

I checked the proportion of responsive transcripts against those that have a BLAST hit.

```
annotation.table.responsive <- annotation.table[which(annotation.table$best.hit.to.nr != "-"), ]

responsive_transcripts_w_BLAST <- length(which(unique(resp.TPM.dt.sub$Transcript) %in% annotation.table

responsive_transcripts_w_BLAST / round(nrow(annotation.table.responsive))
```

```
## [1] 0.0141
```

Compare temperature of maximum expression between species

Probability density function of peak expression for transcripts that differ in expression between *A22* and *Ar*. For this analysis, I used only the transcripts with a significant temperature by species interaction.

```
# reshape data
Ap.df <- data.frame(Transcript = rep(interaction.response.type$Transcript, times = 2),
                    colony = rep(c("ApVT", "AcNC"),
                                 each = length(interaction.response.type$Transcript)),
                    max.val = c(interaction.response.type$A22.max, interaction.response.type$Ar.max))

maxexplot <- ggplot(Ap.df, aes(x=max.val, fill=colony)) +
```

```r
  geom_density(alpha=0.2, position="identity") +
  labs(x = "Temperature of maximum expression", y = "Density") +
  theme(axis.title = element_text(size = rel(2))) +
  theme(axis.text = element_text(size = rel(1.2)))
print(maxexplot)
```

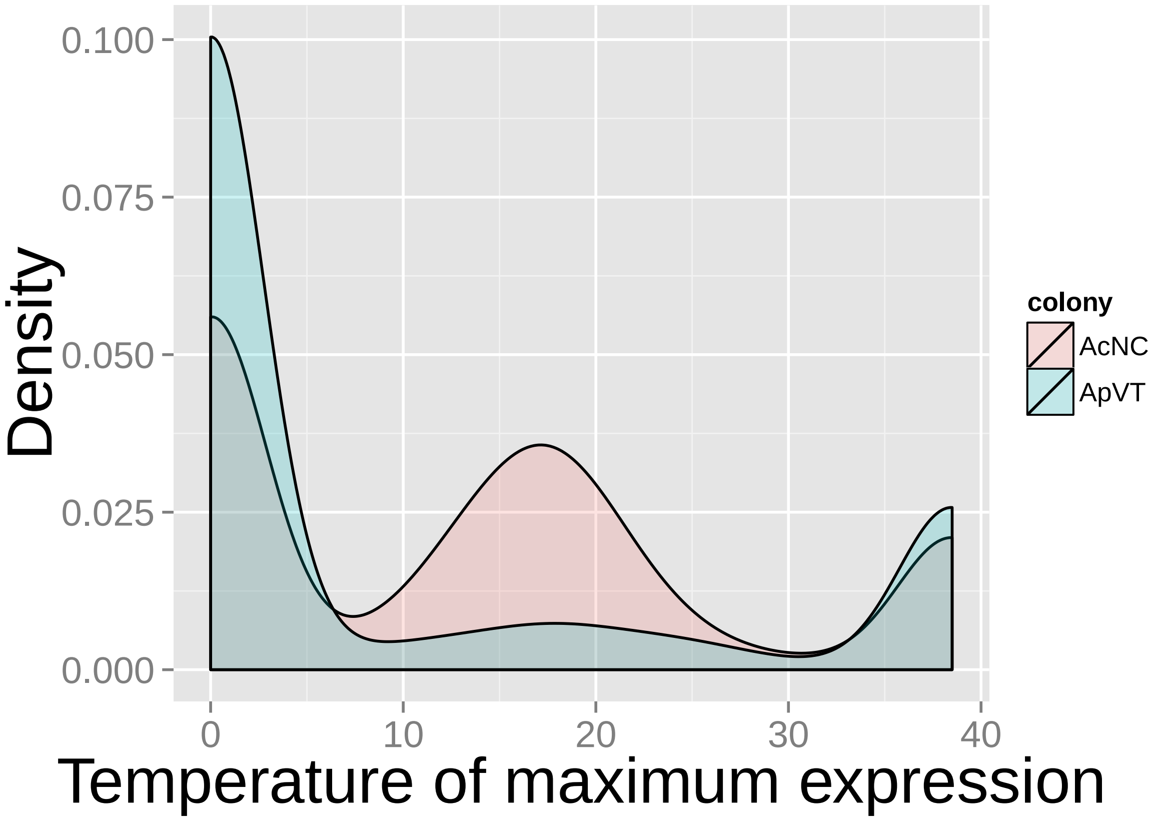


```r
# Figure for presentation
png("results/temp_max_expression.png")
maxexplot + theme(legend.position = "none")
dev.off()
```

```
## pdf
##   2
```

Compare basal expression at optimal temperature for thermally-responsive genes

Genes upregulated in response to thermal stress in one species may have greater basal levels of expression in the other species that experiences those stressful conditions more often. To test this hypothesis, we compared expression levels at the rearing temperature (25C) between the two species for genes in that are either in the 'High' or 'Low' expression group in the other species. Specifically, do genes upregulated at high temperatures in *A22* have greater basal expression at optimal temperatures in *Ar*?

```r
# list of transcripts that are 'high' expressed in A22
A22.high.transcripts.df <- interaction.response.type[which(interaction.response.type$A22.type == "High")
```

```r
# Compare expression at optimum temp (A22.opt) between colonies using t-test
t.test(A22.high.transcripts.df$A22.opt, A22.high.transcripts.df$Ar.opt)
```

```
##
##  Welch Two Sample t-test
##
## data:  A22.high.transcripts.df$A22.opt and A22.high.transcripts.df$Ar.opt
## t = -0.1, df = 400, p-value = 0.9
## alternative hypothesis: true difference in means is not equal to 0
## 95 percent confidence interval:
##  -6794  5844
## sample estimates:
## mean of x mean of y
##      2147      2622
```

```r
boxplot(A22.high.transcripts.df$A22.opt, A22.high.transcripts.df$Ar.opt)
```

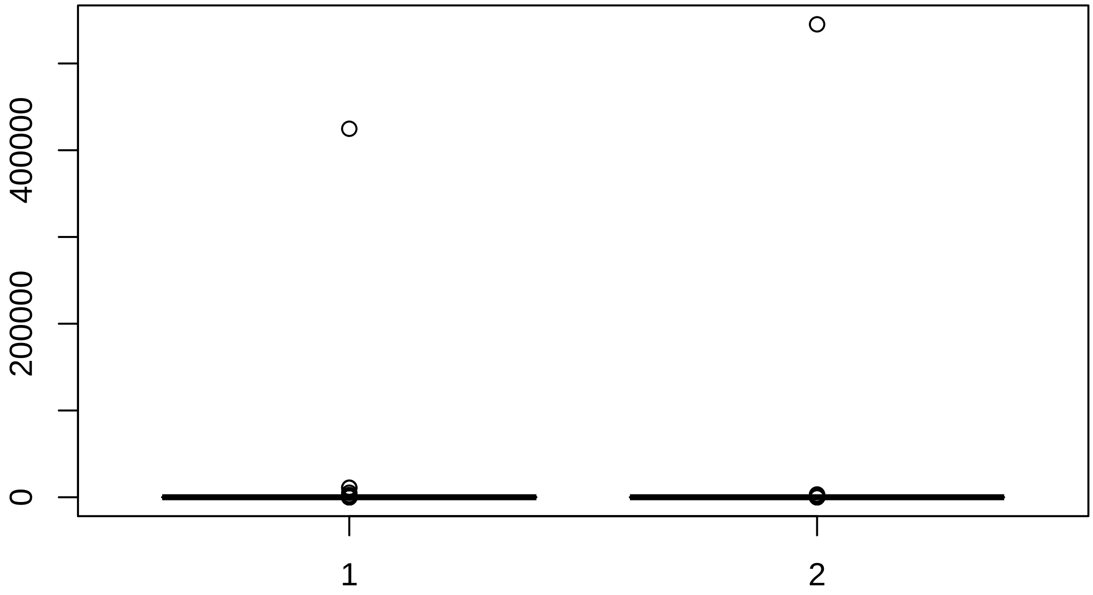


```r
# T test on log-transformed values to control for outliers
t.test(log(A22.high.transcripts.df$A22.opt+1), log(A22.high.transcripts.df$Ar.opt+1))
```

```
##
##  Welch Two Sample t-test
##
## data:  log(A22.high.transcripts.df$A22.opt + 1) and log(A22.high.transcripts.df$Ar.opt + 1)
## t = -3, df = 400, p-value = 0.002
## alternative hypothesis: true difference in means is not equal to 0
## 95 percent confidence interval:
##  -0.856 -0.189
## sample estimates:
## mean of x mean of y
##      1.47      1.99
```

```r
boxplot(log(A22.high.transcripts.df$A22.opt+1), log(A22.high.transcripts.df$Ar.opt+1))
```

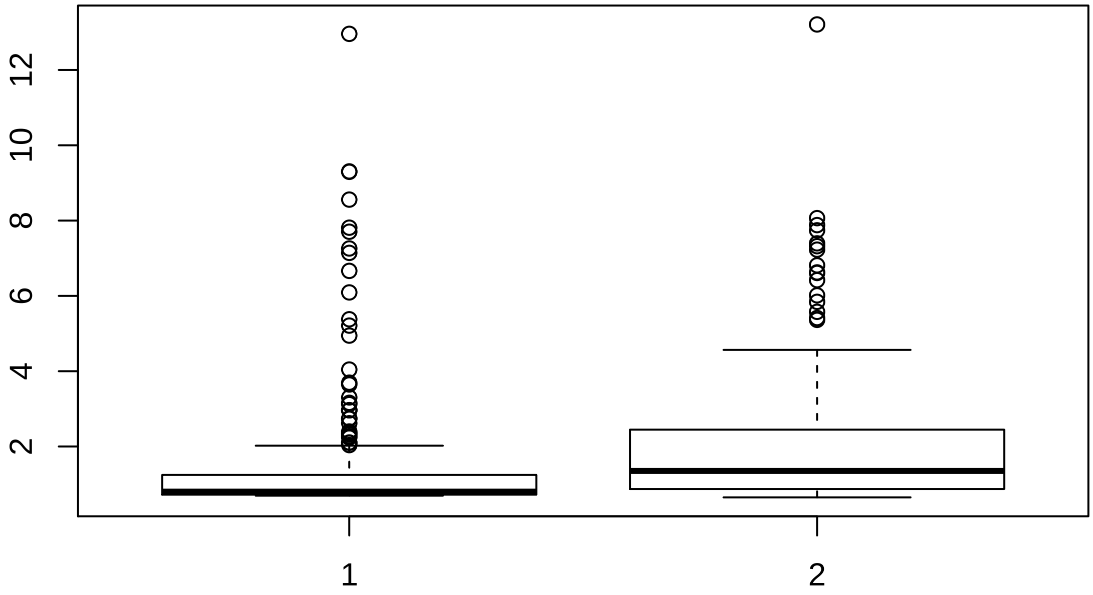

The `t.test` fails to account for the many orders of magnitude difference in expression among transcripts, e.g. non-equal variances. This problem is the key issue in the analysis of differential expression (Bullard, Purdom, Hansen, and Dudoit, 2010; Anders, McCarthy, Chen, Okoniewski, Smyth, Huber, and Robinson, 2013). As my goal is simply to determine if expression is typically greater at optimal temperatures (19.5 C) in *Ar* than *A22* for genes that are up-regulated at high temperatures in *A22*, I use a non-parametric Wilcoxon signed rank-test.

```r
w1 <- wilcox.test(A22.high.transcripts.df$A22.opt, A22.high.transcripts.df$Ar.opt, alternative = "two.s
w1
```

```
##
##  Wilcoxon signed rank test with continuity correction
##
## data:  A22.high.transcripts.df$A22.opt and A22.high.transcripts.df$Ar.opt
## V = 5000, p-value = 2e-12
## alternative hypothesis: true location shift is not equal to 0
## 95 percent confidence interval:
##  -3.31 -1.27
## sample estimates:
## (pseudo)median
##          -1.97
```

Consistent with expectations, there is greater expression at 25C for *Ar* than *A22* transcripts for the set of transcripts that are transcripts that are up-regulated at high temperatures in *A22*. Note that *A22* had the larger library size so if this was due to TPM not correctly accounting for differences in reads between samples, we would expect to see a positive instead of negative value here.

Next I test the converse: do genes up-regulated at low temperatures in *Ar* have greater basal expression near optimal temperatures in *A22*?

```r
# list of transcripts that are 'high' expressed in Ar
Ar.low.transcripts.df <- interaction.response.type[which(interaction.response.type$Ar.type == "Low"), ]

# t-test with log values
t.test(log(Ar.low.transcripts.df$A22.opt+1), log(Ar.low.transcripts.df$Ar.opt+1))
```

```
##
##  Welch Two Sample t-test
##
## data:  log(Ar.low.transcripts.df$A22.opt + 1) and log(Ar.low.transcripts.df$Ar.opt + 1)
## t = -5, df = 1000, p-value = 1e-06
## alternative hypothesis: true difference in means is not equal to 0
## 95 percent confidence interval:
##  -0.371 -0.157
## sample estimates:
## mean of x mean of y
##      1.18      1.45
```

```r
boxplot(log(Ar.low.transcripts.df$A22.opt+1), log(Ar.low.transcripts.df$Ar.opt+1))
```

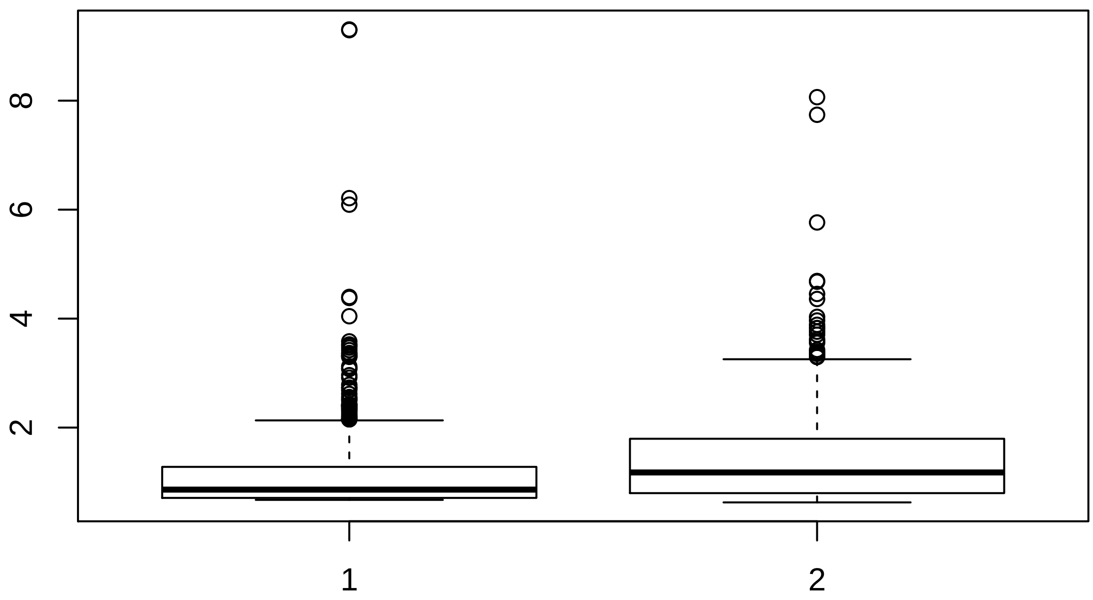


```r
# Wilcoxon signed rank-test
w2 <- wilcox.test(Ar.low.transcripts.df$A22.opt, Ar.low.transcripts.df$Ar.opt, alternative = "two.sided
w2
```

```
##
##  Wilcoxon signed rank test with continuity correction
##
## data:  Ar.low.transcripts.df$A22.opt and Ar.low.transcripts.df$Ar.opt
## V = 40000, p-value <2e-16
## alternative hypothesis: true location shift is not equal to 0
## 95 percent confidence interval:
##  -1.221 -0.729
## sample estimates:
```

```
## (pseudo)median
##         -0.956
```

Counter to expectations, expression at optimal temperatures is also greater in *Ar* than *A22* for transcripts upregulated at low temperatures in *Ar*.

To confirm that there are not sample-level issues, I performed the same comparison using *Intermediate* expressed-genes where I do not expect to see a difference in expression.

```
# list of transcripts that are 'Intermediate' expressed in either colony
Ap.int.transcripts <- interaction.response.type[which(interaction.response.type$Ar.type == "Intermediat
# T test
t.test(log(Ap.int.transcripts$A22.opt+1), log(Ap.int.transcripts$Ar.opt+1))
```

```
##
##  Welch Two Sample t-test
##
## data:  log(Ap.int.transcripts$A22.opt + 1) and log(Ap.int.transcripts$Ar.opt + 1)
## t = -5, df = 1000, p-value = 8e-08
## alternative hypothesis: true difference in means is not equal to 0
## 95 percent confidence interval:
##  -0.512 -0.239
## sample estimates:
## mean of x mean of y
##      1.43      1.80
```

```
# Wilcoxon signed rank-test
w3 <- wilcox.test(Ap.int.transcripts$A22.opt, Ap.int.transcripts$Ar.opt, alternative = "two.sided", pai
w3
```

```
##
##  Wilcoxon signed rank test with continuity correction
##
## data:  Ap.int.transcripts$A22.opt and Ap.int.transcripts$Ar.opt
## V = 70000, p-value <2e-16
## alternative hypothesis: true location shift is not equal to 0
## 95 percent confidence interval:
##  -1.69 -1.02
## sample estimates:
## (pseudo)median
##          -1.31
```

The non-parametric test for both comparisions also finds greater expression in *Ar* than *A22* at the optimal temperature.

To visualize this data, I plot the log ratio of inducibility between species against the log ratio of constitutive expression between species. I do this separately for **High** transcripts in *A. picea* and then **Low** transcripts in *A. carolinensis*.

First, I calculate the degree of expression induction as the proportion difference between the maximum and minimum expression levels for each transcript.

```
# calculate degree of induction
Ap.induction <- ddply(resp.TPM.dt.sub.pred, .(colony, Transcript), summarise,
                      upreg = (max(pTPM) - min(pTPM))/min(pTPM) * 100 )
# cast to wide
Ap.induction.wide <- dcast(Ap.induction, Transcript ~ colony, value.var = "upreg")
names(Ap.induction.wide)[names(Ap.induction.wide)=="A22"] <- "A22.upreg"
names(Ap.induction.wide)[names(Ap.induction.wide)=="Ar"] <- "Ar.upreg"

# merge optimum expression, in interaction.response.type data.frame, with predicted TPM data
Ap.induction.wide <- data.table(Ap.induction.wide)
setkey(Ap.induction.wide, Transcript)
interaction.response.type2 <- Ap.induction.wide[interaction.response.type]
```

I extracted the **High** transcripts for *A. picea* and examined the log ratio of inducibility against the log ratio of constitutive expression in *A. picea* over *A. carolinensis**. According to the *genetic assimilation hypothesis* there should be a negative relationship between these ratios. That is, transcripts with greater constitutive expression in *A. carolinensis* should have greater inducibility in response to** High** temperatures in *A. picea*.

```
interaction.response.type2.A22.high <- interaction.response.type2[interaction.response.type2$Transcript
str(interaction.response.type2.A22.high)
```

```
## Classes 'data.table' and 'data.frame':   215 obs. of  11 variables:
##  $ Transcript: chr  "100709|*|comp147744_c0_seq5" "100996|*|comp149394_c1_seq2" "102136|*|comp139488
##  $ A22.upreg : num  9.08 29.27 13.62 4.77 39.78 ...
##  $ Ar.upreg  : num  157.2 156.5 125.4 73.1 20.3 ...
##  $ A22.max   : num  38.5 38.5 38.5 38.5 34.5 38.5 38.5 38.5 38.5 38.5 ...
##  $ A22.min   : num  0 0.5 0 14.2 0 ...
##  $ A22.opt   : num  1.09 1.53 1.66 1 139.49 ...
##  $ A22.type  : chr  "High" "High" "High" "High" ...
##  $ Ar.max    : num  0 0 19 0 18.5 NA 0 0 0 0 ...
##  $ Ar.min    : num  38.5 38.5 38.5 26.5 38.5 NA 38.5 38.5 38.5 38.5 ...
##  $ Ar.opt    : num  4.56 19.85 6.32 0.95 1.82 ...
##  $ Ar.type   : chr  "Low" "Low" "Intermediate" "Low" ...
##  - attr(*, "sorted")= chr "Transcript"
##  - attr(*, ".internal.selfref")=<externalptr>
```

```
# filter out NotResp transcripts in Ar
interaction.response.type2.A22.high <- interaction.response.type2.A22.high[which(interaction.response.t

# ratio of upregulation in A22 vs Ar
interaction.response.type2.A22.high$A22.Ar.upreg <- interaction.response.type2.A22.high$A22.upreg / int

# ratio of constitutive expression in Ar vs A22
interaction.response.type2.A22.high$A22.Ar.constitutive <- interaction.response.type2.A22.high$A22.opt

# plot!
gg11 <- ggplot(interaction.response.type2.A22.high, aes(x = log(A22.Ar.upreg), y = log(A22.Ar.constitut
  geom_point() +
  geom_smooth(method = "lm") +
  labs(x = expression(paste("log(inducibility ", italic("A. picea / A. carolinensis"), ")")),
       y = expression(paste("log(constitutive expression ", italic("A. picea / A. carolinensis"), ")"))
```

```r
gg11
```

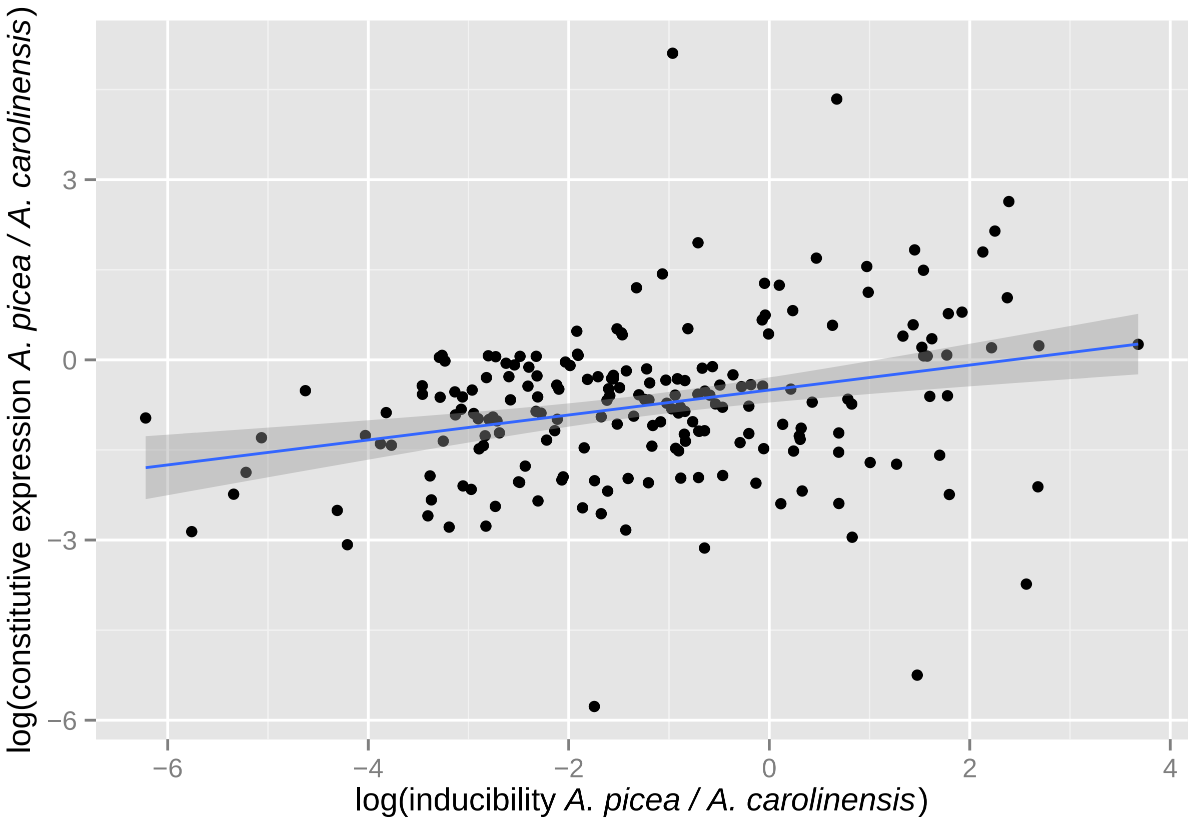


```r
A22.high.lm <- lm(log(A22.Ar.constitutive) ~ log(A22.Ar.upreg), data = interaction.response.type2.A22.hi

summary(A22.high.lm)
```

```
##
## Call:
## lm(formula = log(A22.Ar.constitutive) ~ log(A22.Ar.upreg), data = interaction.response.type2.A22.high
##
## Residuals:
##    Min     1Q Median     3Q    Max
## -5.054 -0.699  0.095  0.621  5.807
##
## Coefficients:
##                   Estimate Std. Error t value Pr(>|t|)
## (Intercept)        -0.5025     0.1059   -4.75  3.9e-06 ***
## log(A22.Ar.upreg)   0.2078     0.0495    4.20  4.0e-05 ***
## ---
## Signif. codes:  0 '***' 0.001 '**' 0.01 '*' 0.05 '.' 0.1 ' ' 1
##
## Residual standard error: 1.27 on 200 degrees of freedom
## Multiple R-squared:  0.0811, Adjusted R-squared:  0.0765
## F-statistic: 17.6 on 1 and 200 DF,  p-value: 0.0000401
```

In contrast to the prediction there is a positive relationship between these ratios. That is, genes with greater upregulation of expression also have greater constitutive expression.

I repeated the above but for **Low** transcripts in *A. carolinensis*.

```
### filter for A. carolinensis *Low* transcripts
interaction.response.type2.Ar.low <- interaction.response.type2[interaction.response.type2$Transcript %
str(interaction.response.type2.Ar.low)
```

```
## Classes 'data.table' and 'data.frame':   538 obs. of  11 variables:
##  $ Transcript: chr  "100709|*|comp147744_c0_seq5" "100996|*|comp149394_c1_seq2" "102562|*|comp125805
##  $ A22.upreg : num  9.08 29.27 0 29.51 0 ...
##  $ Ar.upreg  : num  157.2 156.5 77.6 61.1 278.5 ...
##  $ A22.max   : num  38.5 38.5 NA 0 NA NA 38.5 0 0 NA ...
##  $ A22.min   : num  0 0.5 NA 38.5 NA ...
##  $ A22.opt   : num  1.09 1.53 1 1.17 1 ...
##  $ A22.type  : chr  "High" "High" "NotResp" "Low" ...
##  $ Ar.max    : num  0 0 0 0 0 0 0 0 0 0 ...
##  $ Ar.min    : num  38.5 38.5 26.5 38.5 38.5 38.5 26.5 38.5 38.5 38.5 ...
##  $ Ar.opt    : num  4.562 19.849 0.946 2.053 4.909 ...
##  $ Ar.type   : chr  "Low" "Low" "Low" "Low" ...
##  - attr(*, "sorted")= chr "Transcript"
##  - attr(*, ".internal.selfref")=<externalptr>
```

```
# filter out NotResp transcripts in A22
interaction.response.type2.Ar.low <- interaction.response.type2.Ar.low[which(interaction.response.type2

# ratio of upregulation
interaction.response.type2.Ar.low$Ar.A22.upreg <- interaction.response.type2.Ar.low$Ar.upreg / interact

# ratio of constitutive expression
interaction.response.type2.Ar.low$Ar.A22.constitutive <- interaction.response.type2.Ar.low$Ar.opt / int

gg12 <- ggplot(interaction.response.type2.Ar.low, aes(x = log(Ar.A22.upreg), y = log(Ar.A22.constitutiv
  geom_point() +
  geom_smooth(method = "lm") +
  labs(x = expression(paste("log(inducibility ", italic("A. carolinensis / A. picea"), ")")),
       y = expression(paste("log(constitutive expression ", italic("A. carolinensis / A. picea"), ")"))

gg12
```

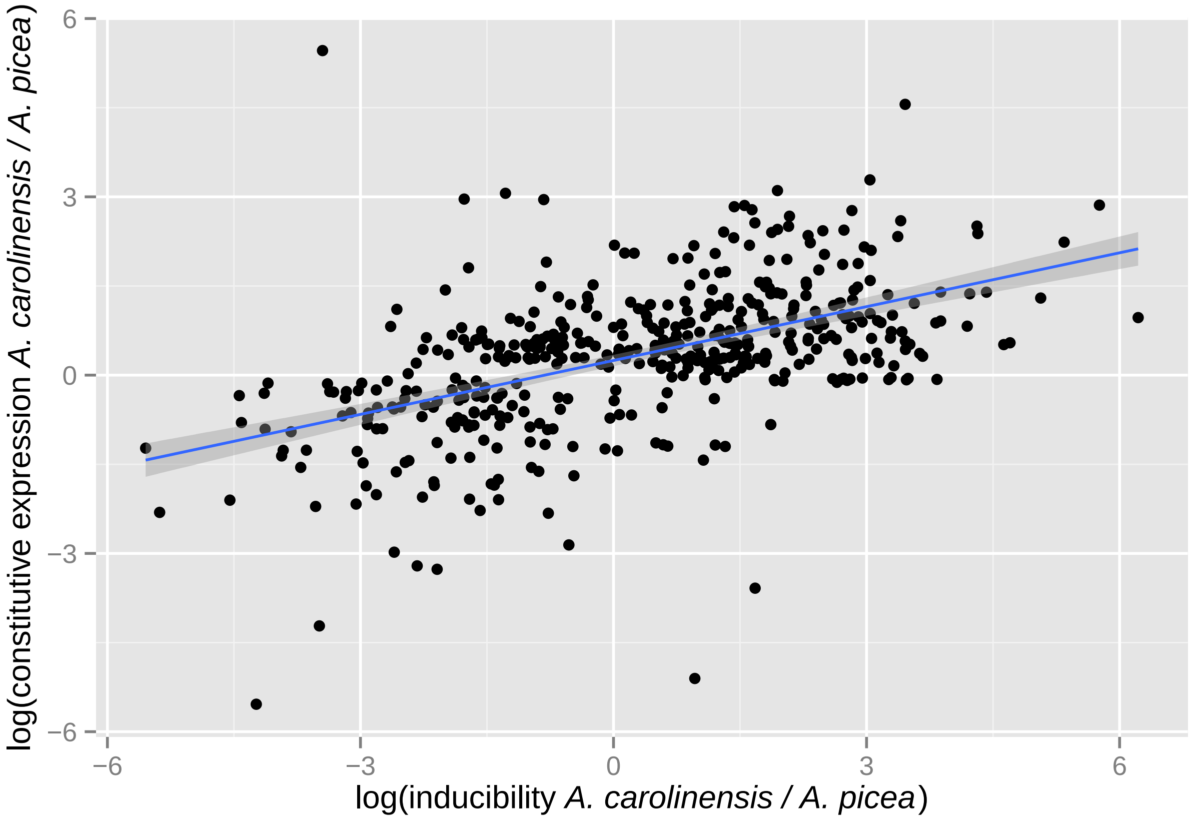


```r
Ar.low.lm <- lm(log(Ar.A22.constitutive) ~ log(Ar.A22.upreg), data = interaction.response.type2.Ar.low)

summary(Ar.low.lm)
```

```
##
## Call:
## lm(formula = log(Ar.A22.constitutive) ~ log(Ar.A22.upreg), data = interaction.response.type2.Ar.low)
##
## Residuals:
##    Min     1Q Median     3Q    Max
## -5.641 -0.507  0.008  0.558  6.257
##
## Coefficients:
##                   Estimate Std. Error t value Pr(>|t|)
## (Intercept)         0.2459     0.0484    5.08  5.4e-07 ***
## log(Ar.A22.upreg)   0.3021     0.0229   13.19  < 2e-16 ***
## ---
## Signif. codes:  0 '***' 0.001 '**' 0.01 '*' 0.05 '.' 0.1 ' ' 1
##
## Residual standard error: 1.04 on 470 degrees of freedom
## Multiple R-squared:  0.27,   Adjusted R-squared:  0.269
## F-statistic:  174 on 1 and 470 DF,  p-value: <2e-16
```

I combine the two above figures into a single figure.

```
# add labels
gg11 <- gg11 + ggtitle('A') + theme(plot.title = element_text(hjust=0))
gg12 <- gg12 + ggtitle('B') + theme(plot.title = element_text(hjust=0))
# grid.arrange
png(file = "results/genetic_assimilation_Fig4.png")
grid.arrange(gg11, gg12, nrow = 1)
dev.off()
```

```
## pdf
##   2
```

As with the previous comparison, I also found no evidence for the genetic assimilation hypothesis.

Compare the critical temperature of transcript upregulation, T_{on}, between species for transcripts induced in response to high or low temperatures

Thermally-responsive genes could also differ in the critical temperature of gene induction. To examine this, I determined the temperature at which each responsive gene had the greatest increase or decrease in expression.

```
# extract TPM data for thermally-responsive transcripts
resp.TPM.dt.sub <- TPM.dt.sub[names(responsive.lms)]
setkey(resp.TPM.dt.sub, Transcript)
str(resp.TPM.dt.sub)
```

```
## Classes 'data.table' and 'data.frame':   45716 obs. of  10 variables:
##  $ Transcript       : chr  "100148|*|comp125464_c0_seq1" "100148|*|comp125464_c0_seq1" "100148|*|comp
##  $ Length           : int  207 207 207 207 207 207 207 207 207 207 ...
##  $ TPM              : num  17.69 0 6.04 0 20.24 ...
##  $ RPKM             : num  20.72 0 9.01 0 25.3 ...
##  $ KPKM             : num  20.72 0 9.01 0 25.3 ...
##  $ EstimatedNumKmers: num  12552 0 4384 0 16552 ...
##  $ EstimatedNumReads: num  156.7 0 54.6 0 206.4 ...
##  $ sample           : chr  "A22-0" "Ar-0" "A22-3" "Ar-3" ...
##  $ val              : num  0 0 3.5 3.5 10.5 10.5 14 14 17.5 17.5 ...
##  $ colony           : Factor w/ 2 levels "A22","Ar": 1 2 1 2 1 2 1 2 1 2 ...
##  - attr(*, ".internal.selfref")=<externalptr>
##  - attr(*, "sorted")= chr "Transcript"
```

```
length(unique(resp.TPM.dt.sub$Transcript))
```

```
## [1] 2078
```

```
# scale transcripts so can compare
resp.TPM.dt.sub[,TPM.scaled:=scale(TPM), by = Transcript]
```

```
##                          Transcript Length    TPM  RPKM  KPKM EstimatedNumKmers
##     1: 100148|*|comp125464_c0_seq1    207 17.692 20.72 20.72             12552
##     2: 100148|*|comp125464_c0_seq1    207  0.000  0.00  0.00                 0
##     3: 100148|*|comp125464_c0_seq1    207  6.042  9.01  9.01              4384
##     4: 100148|*|comp125464_c0_seq1    207  0.000  0.00  0.00                 0
```

```
##      5: 100148|*|comp125464_c0_seq1    207 20.244 25.30 25.30             16552
##    ---
## 45712:   9970|*|comp148838_c0_seq3   1326  3.849  6.50  6.50             13781
## 45713:   9970|*|comp148838_c0_seq3   1326  0.834  1.16  1.16              3993
## 45714:   9970|*|comp148838_c0_seq3   1326  5.851 10.42 10.42             16950
## 45715:   9970|*|comp148838_c0_seq3   1326  1.117  1.53  1.53              6793
## 45716:   9970|*|comp148838_c0_seq3   1326  9.353 17.21 17.21             37338
##        EstimatedNumReads sample  val colony TPM.scaled
##     1:             156.7  A22-0  0.0    A22      2.338
##     2:               0.0   Ar-0  0.0     Ar     -0.687
##     3:              54.6  A22-3  3.5    A22      0.346
##     4:               0.0   Ar-3  3.5     Ar     -0.687
##     5:             206.4 A22-10 10.5    A22      2.775
##    ---
## 45712:             170.4  Ar-31 31.5     Ar      0.182
## 45713:              49.9 A22-35 35.0    A22     -0.701
## 45714:             209.9  Ar-35 35.0     Ar      0.769
## 45715:              84.8 A22-38 38.5    A22     -0.618
## 45716:             461.3  Ar-38 38.5     Ar      1.795
```

```r
# rename colonies
resp.TPM.dt.sub$colony2 <- ifelse(resp.TPM.dt.sub$colony == "A22", "ApVT", "AcNC")
```

For next analyses, I extracted the list of gene names by response type.

```r
interaction.response.type <- as.data.frame(interaction.response.type)
A22.high.transcripts <-  interaction.response.type[which(interaction.response.type$A22.type == "High"),
Ar.high.transcripts <- interaction.response.type[which(interaction.response.type$Ar.type == "High"), "T

A22.low.transcripts <- interaction.response.type[which(interaction.response.type$A22.type == "Low"), "T
Ar.low.transcripts <- interaction.response.type[which(interaction.response.type$Ar.type == "Low"), "Tra

A22.bim.transcripts <- interaction.response.type[which(interaction.response.type$A22.type == "Bimodal")
Ar.bim.transcripts <- interaction.response.type[which(interaction.response.type$Ar.type == "Bimodal"),

A22.int.transcripts <- interaction.response.type[which(interaction.response.type$A22.type == "Intermedi
Ar.int.transcripts <- interaction.response.type[which(interaction.response.type$Ar.type == "Intermediat
```

I calculated T_{on} for *High* genes in each species using the observed (rather than predicted) values for each species as by nature of fitting a quadratic function, the maximum change tends to be at the extremes (38.5 or 0) for the predicted values.

```r
# transcripts expressed at *High* and *Bimodal* temperatures in A22
A22.high.TPM.dt.sub <- resp.TPM.dt.sub.pred[J(union(A22.high.transcripts.df$Transcript, A22.bim.transcr
setkey(A22.high.TPM.dt.sub, Transcript)
str(A22.high.TPM.dt.sub)
```

```
## Classes 'data.table' and 'data.frame':   5137 obs. of  5 variables:
##  $ Transcript: chr  "100148|*|comp125464_c0_seq1" "100148|*|comp125464_c0_seq1" "100148|*|comp125464
##  $ TPM       : num  17.69 6.04 20.24 7.18 4.73 ...
##  $ val       : num  0 3.5 10.5 14 17.5 21 24.5 28 31.5 35 ...
##  $ colony    : Factor w/ 2 levels "A22","Ar": 1 1 1 1 1 1 1 1 1 1 ...
##  $ pTPM      : num  18.01 12.55 7.29 6.09 5.39 ...
```

```
##  - attr(*, ".internal.selfref")=<externalptr>
##  - attr(*, "sorted")= chr "Transcript"
```

```
# make data.frame for results
l1 <- length(unique(A22.high.TPM.dt.sub$Transcript))
A22.high.T_on <- data.frame(Transcript = unique(A22.high.TPM.dt.sub$Transcript), colony = rep("ApVT", le

# loop across transcripts, calculating T_on

for(i in unique(A22.high.TPM.dt.sub$Transcript)) {
    subdf <- A22.high.TPM.dt.sub[i]
    subdf <- subdf[which(subdf$val > 14), ]
    T_on <- subdf[median(which(diff(subdf$TPM) == max(diff(subdf$TPM))))+1, val]
    A22.high.T_on[which(A22.high.T_on$Transcript == i), "T_on"] <- T_on
}

# repeat for Ar
Ar.high.TPM.dt.sub <- resp.TPM.dt.sub.pred[J(union(Ar.high.transcripts, Ar.bim.transcripts), "Ar")]
setkey(Ar.high.TPM.dt.sub, Transcript)
str(Ar.high.TPM.dt.sub)
```

```
## Classes 'data.table' and 'data.frame':   3190 obs. of  5 variables:
##  $ Transcript: chr  "100882|*|comp94953_c0_seq1" "100882|*|comp94953_c0_seq1" "100882|*|comp94953_c0_
##  $ TPM       : num  0 0 0 0.0814 0 ...
##  $ val       : num  0 3.5 10.5 14 17.5 21 24.5 28 31.5 35 ...
##  $ colony    : Factor w/ 2 levels "A22","Ar": 2 2 2 2 2 2 2 2 2 2 ...
##  $ pTPM      : num  0.986 1.002 1.027 1.036 1.043 ...
##  - attr(*, ".internal.selfref")=<externalptr>
##  - attr(*, "sorted")= chr "Transcript"
```

```
l2 <- length(unique(Ar.high.TPM.dt.sub$Transcript))
Ar.high.T_on <- data.frame(Transcript = unique(Ar.high.TPM.dt.sub$Transcript), colony = rep("AcNC", leng

for(i in unique(Ar.high.TPM.dt.sub$Transcript)) {
    subdf <- Ar.high.TPM.dt.sub[i]
    subdf <- subdf[which(subdf$val > 14), ]
    T_on <- subdf[median(which(diff(subdf$TPM) == max(diff(subdf$TPM))))+1, val]
    Ar.high.T_on[which(Ar.high.T_on$Transcript == i), "T_on"] <- T_on
}

# determine if T~on~ is greater in *A22* or *Ar* for *High* genes.

(T_on.high.ttest <- t.test(Ar.high.T_on$T_on, A22.high.T_on$T_on))
```

```
##
##  Welch Two Sample t-test
##
## data:  Ar.high.T_on$T_on and A22.high.T_on$T_on
## t = 0.8, df = 600, p-value = 0.4
## alternative hypothesis: true difference in means is not equal to 0
## 95 percent confidence interval:
##  -0.455  1.129
## sample estimates:
```

```
## mean of x mean of y
##      31.7      31.4
```

This test revealed no differences among species in T_{on} for *High* genes.

I performed the same test comparing T_{on} for *Low* genes.

```
# transcripts expressed at *Low* temperatures in A22
A22.low.TPM.dt.sub <- resp.TPM.dt.sub.pred[J(union(A22.low.transcripts, A22.bim.transcripts), "A22")]
setkey(A22.low.TPM.dt.sub, Transcript)
str(A22.low.TPM.dt.sub)
```

```
## Classes 'data.table' and 'data.frame':   11693 obs. of  5 variables:
##  $ Transcript: chr  "100148|*|comp125464_c0_seq1" "100148|*|comp125464_c0_seq1" "100148|*|comp125464
##  $ TPM       : num  17.69 6.04 20.24 7.18 4.73 ...
##  $ val       : num  0 3.5 10.5 14 17.5 21 24.5 28 31.5 35 ...
##  $ colony    : Factor w/ 2 levels "A22","Ar": 1 1 1 1 1 1 1 1 1 1 ...
##  $ pTPM      : num  18.01 12.55 7.29 6.09 5.39 ...
##  - attr(*, ".internal.selfref")=<externalptr>
##  - attr(*, "sorted")= chr "Transcript"
```

```
# make data.frame for results
l3 <- length(unique(A22.low.TPM.dt.sub$Transcript))
A22.low.T_on <- data.frame(Transcript = unique(A22.low.TPM.dt.sub$Transcript), colony = rep("ApVT", len

# loop across transcripts, calculating T_on
for(i in unique(A22.low.TPM.dt.sub$Transcript)) {
    subdf <- A22.low.TPM.dt.sub[i]
    subdf <- subdf[which(subdf$val < 21), ]
    T_on <- subdf[median(which(diff(subdf$TPM) == max(diff(subdf$TPM))))+1, val]
    A22.low.T_on[which(A22.low.T_on$Transcript == i), "T_on"] <- T_on
}

# repeat for Ar
Ar.low.TPM.dt.sub <- resp.TPM.dt.sub.pred[J(union(Ar.low.transcripts, Ar.bim.transcripts), "A22")]
setkey(Ar.low.TPM.dt.sub, Transcript)
str(Ar.low.TPM.dt.sub)
```

```
## Classes 'data.table' and 'data.frame':   6919 obs. of  5 variables:
##  $ Transcript: chr  "100709|*|comp147744_c0_seq5" "100709|*|comp147744_c0_seq5" "100709|*|comp147744
##  $ TPM       : num  0 0.064 0.159 0.138 0.18 ...
##  $ val       : num  0 3.5 10.5 14 17.5 21 24.5 28 31.5 35 ...
##  $ colony    : Factor w/ 2 levels "A22","Ar": 1 1 1 1 1 1 1 1 1 1 ...
##  $ pTPM      : num  1.03 1.06 1.11 1.12 1.13 ...
##  - attr(*, ".internal.selfref")=<externalptr>
##  - attr(*, "sorted")= chr "Transcript"
```

```
l4 <- length(unique(Ar.low.TPM.dt.sub$Transcript))
Ar.low.T_on <- data.frame(Transcript = unique(Ar.low.TPM.dt.sub$Transcript), colony = rep("AcNC", lengt

for(i in unique(Ar.low.TPM.dt.sub$Transcript)) {
    subdf <- Ar.low.TPM.dt.sub[i]
    subdf <- subdf[which(subdf$val < 21), ]
```

```
    T_on <- subdf[median(which(diff(subdf$TPM) == max(diff(subdf$TPM))))+1, val]
    Ar.low.T_on[which(Ar.low.T_on$Transcript == i), "T_on"] <- T_on
}

(T_on.low.ttest <- t.test(Ar.low.T_on$T_on, A22.low.T_on$T_on))
```

```
##
##  Welch Two Sample t-test
##
## data:  Ar.low.T_on$T_on and A22.low.T_on$T_on
## t = -3, df = 1000, p-value = 0.002
## alternative hypothesis: true difference in means is not equal to 0
## 95 percent confidence interval:
##  -1.080 -0.245
## sample estimates:
## mean of x mean of y
##      12.4      13.1
```

Genes with increased expression at *Low* temperatures are on average turned on 0.2°C higher in *ApVT* than *AcNC*.

Visualize T_{on} for both *Low* and *High* genes on the same plot.

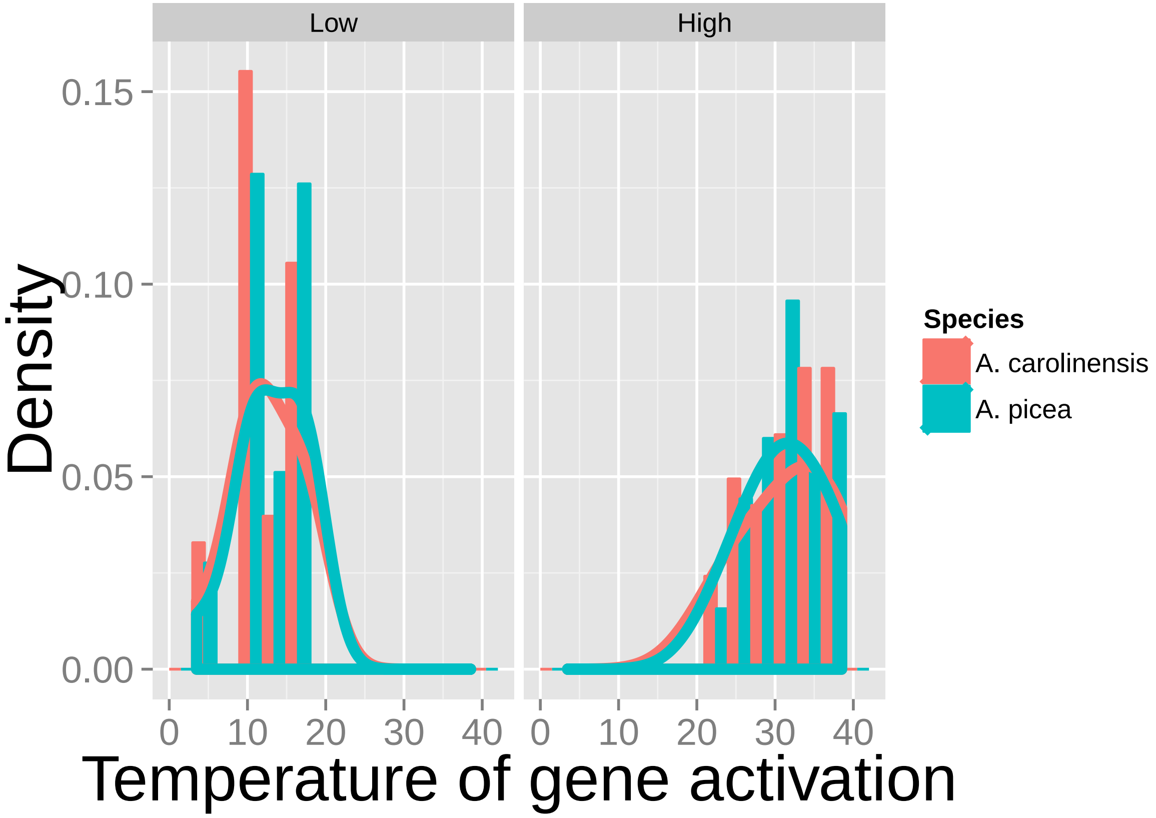

Compare the critical temperature of transcript downregulation, T_{off}, between species for transcripts induced in response to high or low temperatures

This analysis is comparable to the one above, but for the *Intermediate* genes that are downregulated, split into the low and high temperature extremes.

```
# transcripts expressed at *Intermediate* temperatures in A22
A22.int.TPM.dt.sub <- resp.TPM.dt.sub.pred[J(A22.int.transcripts, "A22")]
setkey(A22.int.TPM.dt.sub, Transcript)
str(A22.int.TPM.dt.sub)
```

```
## Classes 'data.table' and 'data.frame':   1815 obs. of  5 variables:
##  $ Transcript: chr  "1010|*|comp147989_c0_seq1" "1010|*|comp147989_c0_seq1" "1010|*|comp147989_c0_se
##  $ TPM       : num  0.544 1.142 0.754 0.831 0.958 ...
##  $ val       : num  0 3.5 10.5 14 17.5 21 24.5 28 31.5 35 ...
##  $ colony    : Factor w/ 2 levels "A22","Ar": 1 1 1 1 1 1 1 1 1 1 ...
##  $ pTPM      : num  1.72 1.78 1.87 1.89 1.9 ...
##  - attr(*, "sorted")= chr "Transcript"
##  - attr(*, ".internal.selfref")=<externalptr>
```

```
# make data.frame for results
l5 <- length(unique(A22.int.TPM.dt.sub$Transcript))
A22.int.T_off <- data.frame(Transcript = unique(A22.int.TPM.dt.sub$Transcript), colony = rep("ApVT", le

# loop across transcripts, calculating T_off at both high and low temperatures
for(i in unique(A22.int.TPM.dt.sub$Transcript)) {
    subdf <- A22.int.TPM.dt.sub[i]

    subdf.high <- subdf[which(subdf$val >= 21), ]
    High <- subdf.high[median(which(diff(subdf.high$TPM) == min(diff(subdf.high$TPM)))), val]
    A22.int.T_off[which(A22.int.T_off$Transcript == i), "High"] <- High

    subdf.low <- subdf[which(subdf$val <= 21), ]
    Low <- subdf.low[median(which(diff(subdf.low$TPM) == min(diff(subdf.low$TPM)))), val]
    A22.int.T_off[which(A22.int.T_off$Transcript == i), "Low"] <- Low
}

# repeat for Ar
Ar.int.TPM.dt.sub <- resp.TPM.dt.sub.pred[J(Ar.int.transcripts, "Ar")]
setkey(Ar.int.TPM.dt.sub, Transcript)
str(Ar.int.TPM.dt.sub)
```

```
## Classes 'data.table' and 'data.frame':   6556 obs. of  5 variables:
##  $ Transcript: chr  "100148|*|comp125464_c0_seq1" "100148|*|comp125464_c0_seq1" "100148|*|comp125464_
##  $ TPM       : num  0 0 0 0 0.0644 ...
##  $ val       : num  0 3.5 10.5 14 17.5 21 24.5 28 31.5 35 ...
##  $ colony    : Factor w/ 2 levels "A22","Ar": 2 2 2 2 2 2 2 2 2 2 ...
##  $ pTPM      : num  0.998 1.003 1.01 1.012 1.012 ...
##  - attr(*, "sorted")= chr "Transcript"
##  - attr(*, ".internal.selfref")=<externalptr>
```

```r
# make data.frame for results
l6 <- length(unique(Ar.int.TPM.dt.sub$Transcript))
Ar.int.T_off <- data.frame(Transcript = unique(Ar.int.TPM.dt.sub$Transcript), colony = rep("AcNC", leng

# loop across transcripts, calculating T_off at both high and low temperatures
for(i in unique(Ar.int.TPM.dt.sub$Transcript)) {
    subdf <- Ar.int.TPM.dt.sub[i]

    subdf.high <- subdf[which(subdf$val >= 21), ]
    High <- subdf.high[median(which(diff(subdf.high$TPM) == min(diff(subdf.high$TPM)))), val]
    Ar.int.T_off[which(Ar.int.T_off$Transcript == i), "High"] <- High

    subdf.low <- subdf[which(subdf$val <= 21), ]
    Low <- subdf.low[median(which(diff(subdf.low$TPM) == min(diff(subdf.low$TPM)))), val]
    Ar.int.T_off[which(Ar.int.T_off$Transcript == i), "Low"] <- Low
}

# compare T_off among species
(T_off.int.high.ttest <- t.test(Ar.int.T_off$High, A22.int.T_off$High))
```

```
##
##  Welch Two Sample t-test
##
## data:  Ar.int.T_off$High and A22.int.T_off$High
## t = 4, df = 300, p-value = 0.0002
## alternative hypothesis: true difference in means is not equal to 0
## 95 percent confidence interval:
##  0.66 2.11
## sample estimates:
## mean of x mean of y
##      28.6      27.2
```

```r
(T_off.int.low.ttest <- t.test(Ar.int.T_off$Low, A22.int.T_off$Low))
```

```
##
##  Welch Two Sample t-test
##
## data:  Ar.int.T_off$Low and A22.int.T_off$Low
## t = 0.5, df = 300, p-value = 0.6
## alternative hypothesis: true difference in means is not equal to 0
## 95 percent confidence interval:
##  -0.781  1.385
## sample estimates:
## mean of x mean of y
##      11.2      10.9
```

As with genes turned on at high temperatures, *A. carolinensis* **Intermediate** genes are down-regulated on at higher temperatures than *A. picea* **Intermediate** genes. Towards low temperatures, the mean temperature of downregulation was not different between species.

Visualize T_{off} for both *Low* and *High* genes on the same plot.

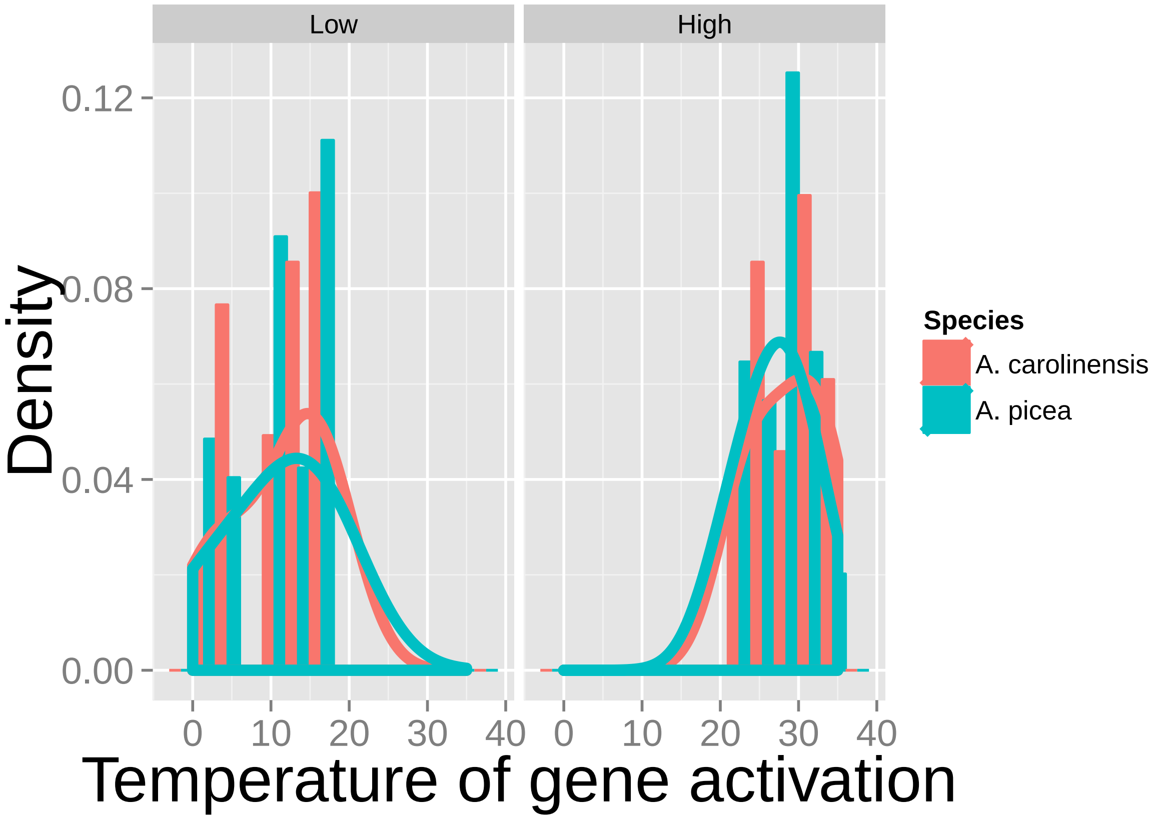

I combined these gene activation comparisons into a single plot.

```
gg2 <- ggplot(Ap.T_on, aes(x = T_on, y = ..density.., colour = Species, group = Species, fill = Species
    facet_grid(. ~ type) +
    geom_histogram(position = "dodge", binwidth = 3) +
    geom_density(adjust = 3, fill = NA, size = 2) +
    labs(x = "Critical temperature of upregulation", y = "Density") +
    theme(legend.position = "none")

gg3 <- ggplot(Ap.T_off, aes(x = T_off, y = ..density.., colour = Species, group = Species, fill = Speci
    facet_grid(. ~ class) +
    geom_histogram(position = "dodge", binwidth = 3) +
    geom_density(adjust = 3, fill = NA, size = 2) +
    labs(x = "Critical temperature of downregulation", y = "Density") +
    theme(legend.position = "bottom", legend.text = element_text(size = 12, face = 3))

# grid.arrange
gg2 <- gg2 + ggtitle('A') + theme(plot.title = element_text(hjust=0))
gg3 <- gg3 + ggtitle('B') + theme(plot.title = element_text(hjust=0))

png("results/crit_temp_regulation_Fig3.png")
grid.arrange(gg2, gg3, nrow = 2, heights = c(.5,.6))
dev.off()
```

```
## pdf
##   2
```

Next, I tested if the mean expression level for all responsive genes differed among colonies.

```r
t.test(log(interaction.response.type$A22.opt), log(interaction.response.type$Ar.opt))
```

```
##
##  Welch Two Sample t-test
##
## data:  log(interaction.response.type$A22.opt) and log(interaction.response.type$Ar.opt)
## t = -7, df = 3000, p-value = 1e-12
## alternative hypothesis: true difference in means is not equal to 0
## 95 percent confidence interval:
##  -0.422 -0.240
## sample estimates:
## mean of x mean of y
##     0.795     1.125
```

```r
# expression level at optimum temp greater in Ar than A22

# non-parametric test
w4 = wilcox.test(interaction.response.type$A22.opt,
                 interaction.response.type$Ar.opt,
                 alternative = "two.sided", paired = TRUE, conf.int = TRUE)


mean.exp <- ddply(resp.TPM.dt.sub.pred, .(colony, Transcript), summarise,
                  mean.TPM = mean(pTPM))

t.test(log(mean.exp[which(mean.exp$colony == "A22"), "mean.TPM"]),
       log(mean.exp[which(mean.exp$colony == "Ar"), "mean.TPM"]))
```

```
##
##  Welch Two Sample t-test
##
## data:  log(mean.exp[which(mean.exp$colony == "A22"), "mean.TPM"]) and log(mean.exp[which(mean.exp$co
## t = -6, df = 3000, p-value = 1e-09
## alternative hypothesis: true difference in means is not equal to 0
## 95 percent confidence interval:
##  -0.372 -0.191
## sample estimates:
## mean of x mean of y
##     0.842     1.123
```

```r
# non-parametric test
w5 = wilcox.test(mean.exp[which(mean.exp$colony == "A22"), "mean.TPM"],
                 mean.exp[which(mean.exp$colony == "Ar"), "mean.TPM"],
                 alternative = "two.sided", paired = TRUE, conf.int = TRUE)


plot(log(mean.exp[which(mean.exp$colony == "A22"), "mean.TPM"]),
     log(mean.exp[which(mean.exp$colony == "Ar"), "mean.TPM"]),
     xlab = "log(expression) A. picea",
     ylab = "log(expression) A. carolinensis")
abline(a=0, b=1)
```

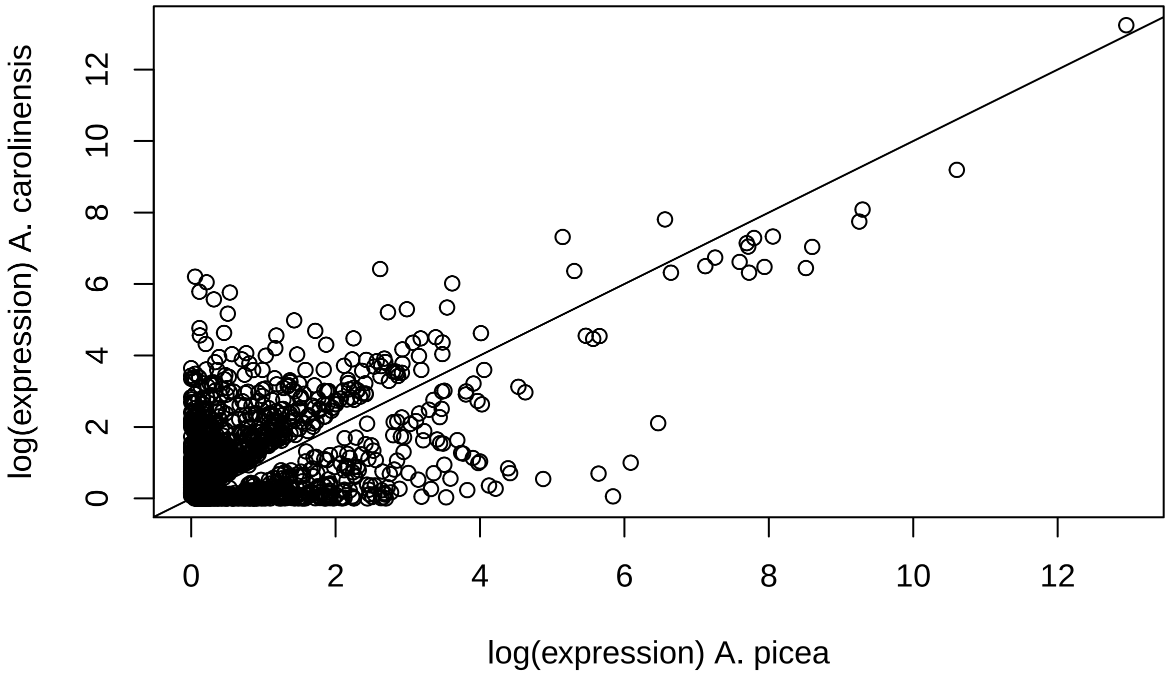

On average, *A. carolinensis* has higher expression than *A. picea*.

Gene set enrichment analysis for thermal-responsive genes

Functional annotation

In the previous section, I identified transcripts that show significant responses in expression. Next, I add gene annotation and ontology information to these transcripts.

```
setkey(annotation.table, Sequence.Name)
signif.transcripts <- data.table(signif.transcripts)
setkey(signif.transcripts, Transcript)
signif.transcripts <- annotation.table[signif.transcripts]
setnames(signif.transcripts, "Sequence.Name", "Transcript")
```

```
responsive.lms.ann.type <- signif.transcripts[names(responsive.lms)]
# combine responsive.lms.ann with "type" information
cols <- c("Transcript", "A22.type", "Ar.type")
subtab <- sig.response.type[, cols, with = FALSE]
setkey(subtab, "Transcript")
responsive.lms.ann.type <- base::merge(responsive.lms.ann.type, subtab, by = "Transcript", all = TRUE)
str(responsive.lms.ann.type)
```

```
## Classes 'data.table' and 'data.frame':   2078 obs. of  17 variables:
##  $ Transcript          : chr  "100148|*|comp125464_c0_seq1" "100636|*|comp3558092_c0_seq1" "100709|*
##  $ sequence.length     : int  207 207 207 207 207 206 1312 206 4232 206 ...
##  $ best.hit.to.nr      : chr  "gi|322798083|gb|EFZ19922.1| hypothetical protein SINV_07083 " "-" "-
##  $ hit.length          : chr  "69" "-" "-" "-" ...
##  $ E.value             : chr  "8.02e-20" "-" "-" "-" ...
##  $ Bit.score           : chr  "101.036156" "-" "-" "-" ...
##  $ GO.Biological.Process: chr  "GO:0006508 proteolysis" "-" "-" "-" ...
```

```
##  $ GO.Cellular.Component: chr  "-" "-" "-" "-" ...
##  $ GO.Molecular.Function: chr  "GO:0004252 serine-type endopeptidase activity" "-" "-" "-" ...
##  $ Enzyme               : chr  "-" "-" "-" "-" ...
##  $ Domain               : chr  "-" "-" "pfam13894 zf-C2H2_4" "-" ...
##  $ annotation.type      : chr  "GO only" "" "Domain only" "" ...
##  $ pval                 : num  2.13e-06 1.54e-07 2.20e-06 1.94e-07 1.15e-18 ...
##  $ adj.r.squared        : num  0.82 0.872 0.82 0.868 0.995 ...
##  $ padj                 : num  1.16e-04 1.46e-05 1.20e-04 1.76e-05 9.38e-15 ...
##  $ A22.type             : chr  "Bimodal" "Low" "High" "Low" ...
##  $ Ar.type              : chr  "Intermediate" "NotResp" "Low" "NotResp" ...
##  - attr(*, "sorted")= chr "Transcript"
##  - attr(*, ".internal.selfref")=<externalptr>
```

```
write.csv(responsive.lms.ann.type, file = paste(resultsdir, "Ap_responsive_genes_", Sys.Date(), ".csv",
```

Proportion of responsive transcripts annotated

Of the responsive transcripts, 49% are annotated, while 51% of all transcripts are annotated.

Candidate gene enrichment

First, I explore of candidate genes with the GO term "response to stress" are enriched in the responsive data set.

```
# GO 'response to stress' hits in responsive transcripts
GO0006950.responsive <- responsive.lms.ann.type[grep("GO:0006950", responsive.lms.ann.type$GO.Biologica

# in high category
GO0006950.responsive[union(with(GO0006950.responsive, grep("High", Ar.type)), with(GO0006950.responsive
```

```
##                   Transcript
## 1: 23441|*|comp114823_c1_seq1
##                                                               best.hit.to.nr A22.type
## 1: gi|443696809|gb|ELT97425.1| hypothetical protein CAPTEDRAFT_194915      High
##    Ar.type
## 1:     Low
```

```
# in low category
GO0006950.responsive[union(with(GO0006950.responsive, grep("Low", Ar.type)), with(GO0006950.responsive,
```

```
##                    Transcript
## 1:  23441|*|comp114823_c1_seq1
## 2: 50934|*|comp3428507_c0_seq1
## 3:  17710|*|comp150271_c3_seq3
##                                                               best.hit.to.nr A22.type
## 1: gi|443696809|gb|ELT97425.1| hypothetical protein CAPTEDRAFT_194915      High
## 2:                                 gi|15010456|gb|AAK77276.1| GH05807p       Low
## 3:        gi|322799248|gb|EFZ20646.1| hypothetical protein SINV_03807       Low
##         Ar.type
## 1:          Low
## 2:          Low
## 3: Intermediate
```

```r
# in bimodal category
GO0006950.responsive[union(with(GO0006950.responsive, grep("Bimodal", Ar.type)), with(GO0006950.responsi
```

```
## Empty data.table (0 rows) of 4 cols: Transcript,best.hit.to.nr,A22.type,Ar.type
```

```r
# in intermediate category
GO0006950.responsive[union(with(GO0006950.responsive, grep("Intermediate", Ar.type)), with(GO0006950.re
```

```
##                   Transcript
## 1: 17710|*|comp150271_c3_seq3
##                                                     best.hit.to.nr A22.type
## 1: gi|322799248|gb|EFZ20646.1| hypothetical protein SINV_03807       Low
##         Ar.type
## 1: Intermediate
```

```r
# Chi-square test to see if 'response to stress' related genes overrepresented in responsive.lms compar
resp.stress.responsive.count <- nrow(responsive.lms.ann.type[grep("GO:0006950", responsive.lms.ann.type$
# GO 'response to stress' hits in all transcripts
resp.stress.all.count <- nrow(annotation.table[grep("GO:0006950", annotation.table$GO.Biological.Proces

GO.stress.table <- matrix(rbind(resp.stress.responsive.count, nrow(responsive.lms.ann.type) - resp.stres

GO.stress.Xsq <- chisq.test(GO.stress.table)
GO.stress.Xsq
```

```
##
##  Pearson's Chi-squared test with Yates' continuity correction
##
## data:  GO.stress.table
## X-squared = 1, df = 1, p-value = 0.3
```

While 3 "response to stress" genes are in the responsive set, this is not enriched compared to the whole transcriptome.

```r
hsp_responsive <- responsive.lms.ann.type[grep("shock", responsive.lms.ann.type$best.hit.to.nr), list(T
hsp_responsive
```

```
## Empty data.table (0 rows) of 4 cols: Transcript,best.hit.to.nr,A22.type,Ar.type
```

```r
hsp_all <- annotation.table[grep("shock", annotation.table$best.hit.to.nr), list(Sequence.Name, best.hi
nrow(hsp_all)
```

```
## [1] 122
```

There are no heat shock protein-related genes in the responsive transcripts.

Gene set enrichment analysis

I used topGO to perform gene set enrichment analysis (GSEA) seperately for each expression type (bimodal, intermediate, high, low). With the GSEA results, I applied the `selectFDR` function to select transcripts with adjusted P < 0.05. For many of the responsive categories, it appeared that there is considerable redundancy in the enriched GO terms. I used the GOSemSim package to determine semantic similarity of enriched GO terms (Yu, Li, Qin, Bo, Wu, and Wang, 2010) and then performed hierarhichal clustering based on this information distance.

For the GSEA, I first created a gene ID to GO term map file.

```
# create geneid2go.map file from FastAnnotator annotationtable.txt
mapname = "results/geneid2go.map"
if(file.exists(mapname)) print("Gene ID to GO term file exists") else{
    geneid2GOmap(as.data.frame(annotation.table), ontology = c("BP", "CC", "MF"), mapname = mapname)
}
```

```
## [1] "Gene ID to GO term file exists"
```

Then I read the map file into memory.

```
# read mappings file
geneID2GO <- readMappings(file = mapname)
str(head(geneID2GO))
```

```
## List of 6
##  $ 0|*|Contig6267              : chr [1:6] "GO:0035335" "GO:0000188" "GO:0006570" "GO:0017017" ...
##  $ 100000|*|comp2663136_c0_seq1: chr ""
##  $ 100001|*|comp3439067_c0_seq1: chr ""
##  $ 100002|*|comp2050457_c0_seq1: chr [1:6] "GO:0006508" "GO:0006412" "GO:0042254" "GO:0005840" ...
##  $ 100003|*|comp2029723_c0_seq1: chr [1:3] "GO:0015074" "GO:0006310" "GO:0003677"
##  $ 100004|*|comp131141_c1_seq1 : chr ""
```

GSEA for thermally-responsive transcripts

Using this gene2GO map file, I performed GSEA for:

1) all responsive transcripts

```
# create geneList. note that NA values cause problems with topGO
# so set any NA to 1 as need to retain for GO analysis
Ap.geneList <- RxNpval$padj
Ap.geneList[which(is.na(Ap.geneList))] <- 1
stopifnot(length(which(is.na(Ap.geneList))) == 0)
names(Ap.geneList) <- RxNpval$Transcript
str(Ap.geneList)

# Function to select genes with adjusted P-value < 0.05
selectFDR <- function(padj) {
    return(padj < 0.05)
}

# create topGOdata object
```

```
Ap.BP.GOdata <- new("topGOdata",
                  description = "BP gene set analysis", ontology = "BP",
                  allGenes = Ap.geneList,
                  geneSel = selectFDR,
                  nodeSize = 10,
                  annot = annFUN.gene2GO, gene2GO = geneID2GO)

#Ap.BP.GOdata

# perform enrichment analysis using parentchild method
Ap.BP.resultParentChild <- runTest(Ap.BP.GOdata, statistic = 'fisher', algorithm = 'parentchild')
Ap.BP.resultParentChild

# table results
Ap.BP.ResTable <- GenTable(Ap.BP.GOdata, parentchild = Ap.BP.resultParentChild, topNodes = 131)
Ap.BP.ResTable
```

As the molecular processes involved in cold and hot temperature tolerance are different, I performed GSEA separately for each response type and compiled results in a single table.

```
sig.response.type <- as.data.frame(sig.response.type)
## Genes with *High* expression in both colonies
Ap.high <- sig.response.type[which(sig.response.type$Ar.type == "High" & sig.response.type$A22.type ==
Ap.geneList.high <- rep(0, length = length(Ap.geneList))
names(Ap.geneList.high) <- names(Ap.geneList)
Ap.geneList.high[(which(names(Ap.geneList.high) %in% Ap.high))] <- 1
# check correct number of values set to 1
table(Ap.geneList.high)
# run GSEA
Ap.high.gsea <- gsea(genelist = Ap.geneList.high, geneID2GO = geneID2GO, plotpath = NA)
```

A single GO term, *c("GO:0044767", "GO:0044700", "GO:0044707") c("single-organism developmental process", "single organism signaling", "single-multicellular organism process")*, is enriched for **High** genes in both species.

```
## Genes with *Low* expression in both colonies
Ap.low <- sig.response.type[which(sig.response.type$Ar.type == "Low" & sig.response.type$A22.type == "L
Ap.geneList.low <- rep(0, length = length(Ap.geneList))
names(Ap.geneList.low) <- names(Ap.geneList)
Ap.geneList.low[(which(names(Ap.geneList.low) %in% Ap.low))] <- 1
# check correct number of values set to 1
table(Ap.geneList.low)
# Run GSEA
Ap.low.gsea <- gsea(genelist = Ap.geneList.low, geneID2GO = geneID2GO)
```

```
## Warning in getSigGroups(object, test.stat): No enrichment can pe performed -
## there are no feasible GO terms!
```

The GO terms, *mitochondrial genome maintenance*, are enriched for **Low** genes in both species.

```r
# Genes with *Bimodal* expression in both colonies
Ap.bim <- sig.response.type[which(sig.response.type$A22.type == "Bimodal" & sig.response.type$Ar.type ==
# create gene list, setting value to 1 for "bim" transcripts
Ap.geneList.bim <- rep(0, length = length(Ap.geneList))
names(Ap.geneList.bim) <- names(Ap.geneList)
Ap.geneList.bim[(which(names(Ap.geneList.bim) %in% Ap.bim))] <- 1
# check correct number of values set to 1
table(Ap.geneList.bim)
```

```
## Ap.geneList.bim
##     0
## 98186
```

```r
# Run GSEA
Ap.bim.gsea <- gsea(genelist = Ap.geneList.bim, geneID2GO = geneID2GO)
```

```
##
## Building most specific GOs ..... ( 5429 GO terms found. )
##
## Build GO DAG topology .......... ( 9145 GO terms and 21227 relations. )
##
## Annotating nodes ............... ( 30235 genes annotated to the GO terms. )
##
##           -- Parent-Child Algorithm --
##
##       the algorithm is scoring 0 nontrivial nodes
##       parameters:
##           test statistic:  fisher : joinFun = union
```

```
## Warning in getSigGroups(object, test.stat): No enrichment can pe performed -
## there are no feasible GO terms!
```

The GO terms, *mitochondrial genome maintenance*, are enriched for **Bimodal** genes in both species.

```r
# genes with *Intermediate* expression in both colonies
Ap.int <- sig.response.type[which(sig.response.type$A22.type == "Intermediate" & sig.response.type$Ar.ty
# create gene list, setting value to 1 for "int" transcripts
Ap.geneList.int <- rep(0, length = length(Ap.geneList))
names(Ap.geneList.int) <- names(Ap.geneList)
Ap.geneList.int[(which(names(Ap.geneList.int) %in% Ap.int))] <- 1
# check correct number of values set to 1
table(Ap.geneList.int)
# run GSEA
Ap.int.gsea <- gsea(genelist = Ap.geneList.int, geneID2GO = geneID2GO)
```

```
## Warning in getSigGroups(object, test.stat): No enrichment can pe performed -
## there are no feasible GO terms!
```

The GO terms, *mitochondrial genome maintenance*, are enriched for **Intermediate** genes in both species.

GSEA for genes that are **Bimodal** in *A. picea* and downregulated (**Intermediate**) in *A. carolinensis*.

```
A22.bim.Ar.int <- sig.response.type[which(sig.response.type$Ar.type == "Intermediate" & sig.response.typ
A22.bim.Ar.int.geneList <- rep(0, length = length(Ap.geneList))
names(A22.bim.Ar.int.geneList) <- names(Ap.geneList)
A22.bim.Ar.int.geneList[(which(names(A22.bim.Ar.int.geneList) %in% A22.bim.Ar.int))] <- 1
# check correct number of values set to 1
table(A22.bim.Ar.int.geneList)
# run GSEA
A22.bim.Ar.int.gsea <- gsea(genelist = A22.bim.Ar.int.geneList, geneID2GO = geneID2GO, plotpath = NA)
```

The GO terms, *autophagy, carbohydrate phosphorylation*, are enriched for genes that are **Bimodal** in *A. picea* and downregulated (**Intermediate**) in *A. carolinensis*.

GSEA for each functional type in each species

A. picea

```
# A22 'High' genes
A22.geneList.high <- rep(0, length = length(Ap.geneList))
names(A22.geneList.high) <- names(Ap.geneList)
A22.geneList.high[(which(names(A22.geneList.high) %in% A22.high.transcripts))] <- 1
A22.high.gsea <- gsea(genelist = A22.geneList.high, geneID2GO = geneID2GO)

# A22 'Low' genes
A22.geneList.low <- rep(0, length = length(Ap.geneList))
names(A22.geneList.low) <- names(Ap.geneList)
A22.geneList.low[(which(names(A22.geneList.low) %in% A22.low.transcripts))] <- 1
A22.low.gsea <- gsea(genelist = A22.geneList.low, geneID2GO = geneID2GO)

# A22 'Bim' genes
A22.geneList.bim <- rep(0, length = length(Ap.geneList))
names(A22.geneList.bim) <- names(Ap.geneList)
A22.geneList.bim[(which(names(A22.geneList.bim) %in% A22.bim.transcripts))] <- 1
A22.bim.gsea <- gsea(genelist = A22.geneList.bim, geneID2GO = geneID2GO)

# A22 'Int' genes
A22.geneList.int <- rep(0, length = length(Ap.geneList))
names(A22.geneList.int) <- names(Ap.geneList)
A22.geneList.int[(which(names(A22.geneList.int) %in% A22.int.transcripts))] <- 1
A22.int.gsea <- gsea(genelist = A22.geneList.int, geneID2GO = geneID2GO)
```

A. carolinensis

```
# Ar 'High' genes
Ar.geneList.high <- rep(0, length = length(Ap.geneList))
names(Ar.geneList.high) <- names(Ap.geneList)
Ar.geneList.high[(which(names(Ar.geneList.high) %in% Ar.high.transcripts))] <- 1
Ar.high.gsea <- gsea(genelist = Ar.geneList.high, geneID2GO = geneID2GO)

# Ar 'Low' genes
Ar.geneList.low <- rep(0, length = length(Ap.geneList))
names(Ar.geneList.low) <- names(Ap.geneList)
Ar.geneList.low[(which(names(Ar.geneList.low) %in% Ar.low.transcripts))] <- 1
```

```r
Ar.low.gsea <- gsea(genelist = Ar.geneList.low, geneID2GO = geneID2GO)

# Ar 'Bim' genes
Ar.geneList.bim <- rep(0, length = length(Ap.geneList))
names(Ar.geneList.bim) <- names(Ap.geneList)
Ar.geneList.bim[(which(names(Ar.geneList.bim) %in% Ar.bim.transcripts))] <- 1
Ar.bim.gsea <- gsea(genelist = Ar.geneList.bim, geneID2GO = geneID2GO)

# Ar 'Int' genes
Ar.geneList.int <- rep(0, length = length(Ap.geneList))
names(Ar.geneList.int) <- names(Ap.geneList)
Ar.geneList.int[(which(names(Ar.geneList.int) %in% Ar.int.transcripts))] <- 1
Ar.int.gsea <- gsea(genelist = Ar.geneList.int, geneID2GO = geneID2GO)
```

Combined gene set enrichment analysis for each species and category into single table.

```r
# combine into single table
A22.high.gsea$Type <- "High"
A22.low.gsea$Type <- "Low"
A22.int.gsea$Type <- "Intermediate"
A22.bim.gsea$Type <- "Bimodal"
A22.gsea <- rbind(A22.high.gsea, A22.low.gsea, A22.int.gsea, A22.bim.gsea)
A22.gsea$Species <- "ApVT"

Ar.high.gsea$Type <- "High"
Ar.low.gsea$Type <- "Low"
Ar.bim.gsea$Type <- "Bimodal"
Ar.int.gsea$Type <- "Intermediate"
Ar.gsea <- rbind(Ar.high.gsea, Ar.low.gsea, Ar.int.gsea, Ar.bim.gsea)
Ar.gsea$Species <- "AcNC"

# combine
Ap.gsea.by.species <- rbind(A22.gsea, Ar.gsea)
# reorder
Ap.gsea.by.species <- Ap.gsea.by.species[,c("Species", "Type", "GO.ID", "Term", "Annotated", "Significa
colnames(Ap.gsea.by.species)[8] <- "P"

write.csv(Ap.gsea.by.species, file = paste(resultsdir, "Ap_gsea_by_species", Sys.Date(), ".csv", sep =
```

Shiny interactive web-app

To assist visualization of specific transcripts, I made a interactive web-app using the shiny package. The scripts for this app are in the sub-directory `.\ApRxN-shinyapp`. The web app is available at https://johnsg.shinyapps.io/ApRxN-shinyapp/

Export data for interactive shiny app.

Session information

```r
save.image()
```

```
## Warning in save(list = names(.GlobalEnv), file = outfile, version = version, :
## 'package:R.utils' may not be available when loading
```

```r
sessionInfo()
```

```
## R version 3.2.2 (2015-08-14)
## Platform: x86_64-apple-darwin13.4.0 (64-bit)
## Running under: OS X 10.10.5 (Yosemite)
##
## locale:
## [1] en_US.UTF-8/en_US.UTF-8/en_US.UTF-8/C/en_US.UTF-8/en_US.UTF-8
##
## attached base packages:
##  [1] stats4    parallel  grid      stats     graphics  grDevices utils
##  [8] datasets  methods   base
##
## other attached packages:
##  [1] GOSemSim_1.24.1      Rgraphviz_2.10.0     BiocInstaller_1.18.4
##  [4] topGO_2.20.0         SparseM_1.6          GO.db_3.1.2
##  [7] RSQLite_1.0.0        DBI_0.3.1            AnnotationDbi_1.30.1
## [10] GenomeInfoDb_1.2.5   IRanges_2.0.1        S4Vectors_0.4.0
## [13] Biobase_2.26.0       graph_1.44.1         BiocGenerics_0.14.0
## [16] gridExtra_0.9.1      coin_1.0-24          survival_2.38-3
## [19] RColorBrewer_1.1-2   reshape2_1.4.1       xtable_1.7-4
## [22] MASS_7.3-43          plyr_1.8.1           RCurl_1.95-4.6
## [25] bitops_1.0-6         data.table_1.9.4     stringr_0.6.2
## [28] pander_0.5.1         knitcitations_1.0.6  knitr_1.9
## [31] ggplot2_1.0.1        R.utils_2.0.0        R.oo_1.19.0
## [34] R.methodsS3_1.7.0
##
## loaded via a namespace (and not attached):
##  [1] modeltools_0.2-21 splines_3.2.2     lattice_0.20-33   colorspace_1.2-6
##  [5] htmltools_0.2.6   yaml_2.1.13       chron_2.3-45      XML_3.98-1.1
##  [9] munsell_0.4.2     gtable_0.1.2      mvtnorm_1.0-2     codetools_0.2-14
## [13] memoise_0.2.1     evaluate_0.6      proto_0.3-10      Rcpp_0.11.5
## [17] scales_0.2.4      formatR_1.1       digest_0.6.8      RJSONIO_1.3-0
## [21] bibtex_0.4.0      tools_3.2.2       RefManageR_0.8.45 lubridate_1.3.3
## [25] rmarkdown_0.5.1   httr_0.6.1
```

References

[1] S. Anders, D. J. McCarthy, Y. Chen, et al. "Count-based differential expression analysis of RNA sequencing data using R and Bioconductor". In: *Nat Protoc* 8.9 (Aug. 2013), pp. 1765-1786. DOI: 10.1038/nprot.2013.099. .

[2] J. H. Bullard, E. Purdom, K. D. Hansen, et al. "Evaluation of statistical methods for normalization and differential expression in mRNA-Seq experiments". In: *BMC Bioinformatics* 11.1 (2010), p. 94. DOI: 10.1186/1471-2105-11-94. .

[3] M. G. Grabherr, B. J. Haas, M. Yassour, et al. "Full-length transcriptome assembly from RNA-Seq data without a reference genome". In: *Nat Biotechnol* 29.7 (May. 2011), pp. 644-652. DOI: 10.1038/nbt.1883. .

[4] X. Huang. "CAP3: A DNA Sequence Assembly Program". In: *Genome Research* 9.9 (Sep. 1999), pp. 868-877. DOI: 10.1101/gr.9.9.868. .

[5] B. Li, V. Ruotti, R. M. Stewart, et al. “RNA-Seq gene expression estimation with read mapping uncertainty”. In: *Bioinformatics* 26.4 (Dec. 2009), pp. 493-500. DOI: 10.1093/bioinformatics/btp692. .

[6] M. Lohse, A. M. Bolger, A. Nagel, et al. “RobiNA: a user-friendly, integrated software solution for RNA-Seq-based transcriptomics”. In: *Nucleic Acids Research* 40.W1 (Jun. 2012), pp. W622-W627. DOI: 10.1093/nar/gks540. .

[7] D. Lubertazzi. " The Biology and Natural History of Aphaenogaster rudis “. In: *Psyche: A Journal of Entomology* 2012 (2012), pp. 1-11. DOI: 10.1155/2012/752815. .

[8] Y. Yang and S. A. Smith. “Optimizing de novo assembly of short-read RNA-seq data for phylogenomics”. In: *BMC Genomics* 14.1 (2013), p. 328. DOI: 10.1186/1471-2164-14-328. .

[9] G. Yu, F. Li, Y. Qin, et al. “GOSemSim: an R package for measuring semantic similarity among GO terms and gene products”. In: *Bioinformatics* 26.7 (Feb. 2010), pp. 976-978. DOI: 10.1093/bioinformatics/btq064. .
